# Protection of mice deficient in mature B cells from West Nile virus infection by passive and active immunization

**DOI:** 10.1371/journal.ppat.1006743

**Published:** 2017-11-27

**Authors:** Daniela Giordano, Kevin E. Draves, Lucy B. Young, Kelsey Roe, Marianne A. Bryan, Christiane Dresch, Justin M. Richner, Michael S. Diamond, Michael Gale, Edward A. Clark

**Affiliations:** 1 Department of Immunology, University of Washington, Seattle, Washington, United States of America; 2 Departments of Medicine, Molecular Microbiology, Pathology and Immunology, Washington University School of Medicine, St Louis, Missouri, United States of America; 3 The Center for Human Immunology and Immunotherapy Programs, Washington University School of Medicine, St Louis, Missouri, United States of America; 4 Center for Innate Immunity and Immune Disease, University of Washington, Seattle, Washington, United States of America; St. Jude Children's Research Hospital, UNITED STATES

## Abstract

B cell activating factor receptor (BAFFR)^-/-^ mice have a profound reduction in mature B cells, but unlike μMT mice, they have normal numbers of newly formed, immature B cells. Using a West Nile virus (WNV) challenge model that requires antibodies (Abs) for protection, we found that unlike wild-type (WT) mice, BAFFR^-/-^ mice were highly susceptible to WNV and succumbed to infection within 8 to 12 days after subcutaneous virus challenge. Although mature B cells were required to protect against lethal infection, infected BAFFR^-/-^ mice had reduced WNV E-specific IgG responses and neutralizing Abs. Passive transfer of immune sera from previously infected WT mice rescued BAFFR^-/-^ and fully B cell-deficient μMT mice, but unlike μMT mice that died around 30 days post-infection, BAFFR^-/-^ mice survived, developed WNV-specific IgG Abs and overcame a second WNV challenge. Remarkably, protective immunity could be induced in mature B cell-deficient mice. Administration of a WNV E-anti-CD180 conjugate vaccine 30 days prior to WNV infection induced Ab responses that protected against lethal infection in BAFFR^-/-^ mice but not in μMT mice. Thus, the immature B cells present in BAFFR^-/-^ and not μMT mice contribute to protective antiviral immunity. A CD180-based vaccine may promote immunity in immunocompromised individuals.

## Author summary

Many individuals including infants, the elderly and the immunocompromised do not develop protective immunity after immunization with current vaccines. Thus, new vaccine strategies are needed to overcome underlying immune deficiencies and mediate protection. In this study, we examined whether it was possible to induce protective immunity even in BAFFR-deficient mice that genetically lack mature B cells but still make immature B cells. We infected mice with the flavivirus, West Nile virus (WNV), since elderly and immunocompromised individuals are at greatest risk to develop severe neurological disease with this virus. As expected, the BAFFR-deficient mice died within 12 days of infection, similar to mice lacking all B cells. Unexpectedly, and unlike mice missing all B cells, BAFFR-deficient mice could be protected by passively administering immune sera or with a vaccine that can program immature B cells to make antibodies. Thus, immature B cells can play a role in protecting individuals from WNV infection, and accordingly, it is possible to design vaccines that activate these cells to develop protective immunity. These findings may be relevant for developing vaccines that can protect infants, the elderly and immunocompromised patients.

## Introduction

The B cell-activating factor (BAFF, also known as BLyS) is a TNF superfamily member that has a crucial role in B cell homeostasis, survival and maturation [1,2,3,4,5]. BAFF is produced by monocytes (MO), macrophages (Mϕ), DCs and neutrophils (Nph) and binds to three receptors: BAFFR (BR3; TNFRSF13C), transmembrane activator and calcium modulator and cyclophilin ligand interactor (TACI; TNFRSF13B) and B-cell maturation antigen (BCMA; TNFRSF17) [2,6,7]. TACI and BCMA both are expressed at later stages of B cell differentiation on follicular (FO) and marginal zone (MZ) B cells and upon antigen (Ag) encounter, are expressed on plasma cells (PCs) and memory B cells (MBCs). They are not required for mature B cell development [2,7]; in contrast, BAFFR is essential for differentiation and survival of mature B cells. It is expressed at low levels in newly formed bone marrow (BM) B cells and splenic transitional 1 (T1) B cells and at higher levels on splenic transitional 2 (T2), FO and MZ B cells [3,8]. Consistent with this pattern of expression, a deficiency of BAFFR blocks the transition from newly formed T1 to T2 B cells, resulting in an almost complete reduction of T2, FO and MZ B cells [9]. Consequently, BAFFR^-/-^ mice have significantly reduced Ag-specific Ab responses after immunization with T cell-dependent (TD) and some T cell-independent (TI) Ags [9], but have normal Ab responses to TI-2 Ags [10]. BAFFR signaling on T cells also is a mediator of BAFF-dependent co-stimulatory T-cell responses [11].

Because they lack mature B cells, BAFFR^-/-^ mice are an appropriate model of humoral immunodeficiency. However, few studies have addressed the role of BAFFR during viral infection. After infection with Friend leukemia virus, BAFFR^-/-^ mice displayed increased and persistent viremia as well as decreased and delayed neutralizing Ab (nAb) responses [12]. In this study, the number of infected splenic B cells was higher in BAFFR^-/-^ than WT mice. In contrast, BAFFR^-/-^ mice infected with the murine gamma herpesvirus 4 (MuHV-4) had decreased viral titers in lymphoid tissues and defects in B cell maturation, GC formation and Ab responses [13]. BAFFR^-/-^ mice also had delayed nAb responses and succumbed to infection with vesicular stomatitis virus [14]. Thus, although deficiency of BAFFR consistently impaired Ag-specific Ab responses, its absence can lead to increased or reduced viral dissemination and differently affects pathogenesis depending on the virus.

Here, we examined the susceptibility of BAFFR^-/-^ mice to pathogenic West Nile Virus (WNV) infection. WNV is a neurotropic flavivirus that together with other flaviviruses (e.g., Dengue, Zika, yellow fever, and Japanese encephalitis viruses) poses a significant threat to human health [15,16]. Epidemiological analyses have shown that elderly and immunocompromised individuals are at greatest risk to develop severe neurological disease after WNV infection [17,18]. As there are no approved human vaccines or therapies for WNV infection, it is important to investigate factors involved in host susceptibility or resistance to disease [15,18]. WNV pathogenesis is characterized by three phases: an early phase after the bite of an infected mosquito, infection of the skin and local spread to the draining lymph nodes, followed by viral dissemination to peripheral organs, and then a later stage of invasion of the central nervous system (CNS) [15]. The innate immune response controls WNV at the early phase, whereas adaptive humoral and cellular immunity contain virus spread and organs damage at later stages [15,19]. Both CD8^+^ and CD4^+^ T cells are required for protection against WNV [20,21]. CD8^+^ T cells help to eliminate viral infection in the spleen and CNS [20] whereas CD4^+^ T cells prime WNV-specific IgM and IgG responses sustain CD8^+^ T cell responses within the CNS, and have independent protective effector functions [21]. B cell responses and humoral immunity also are essential for protection from WNV infection [17]. B cell- and Ab- deficient (μMT) mice succumb to WNV infection but can be protected transiently by passive transfer of immune sera [22]. While both IgM and IgG Abs can protect from WNV lethal infection, the production of an early neutralizing IgM response controls viremia and triggers a protective IgG response that limits virus spread in the CNS [22,23]. Given the importance of humoral responses several studies have focused on understanding B cell responses and the mechanisms of antibody protection to WNV (reviewed in [24]).

Vaccination strategies that boost WNV-specific Ab production in particular may be useful to help immunocompromised individuals at risk of developing severe WNV infection. The CD180 (RP105) receptor, a pattern recognition receptor related to TLR4, is expressed by B cells and DCs [25,26], and is an attractive vaccine target. Previously, we showed that targeting antigen (Ag) to CD180 by conjugating the Ag to an anti-CD180 Ab induces rapid, strong and persistent Ag-specific Ab responses [27]. CD180 Ag targeting also induced strong IgG responses against model Ag in BAFFR^-/-^ mice and could increase B cell numbers rapidly including T1 and FO B cells in WT mice [27].

In this study we found that after a subcutaneous (s.c.) inoculation with WNV, BAFFR^-/-^ mice succumbed to infection and had reduced and delayed WNV-specific Ab responses; all BAFFR^-/-^ mice had higher viral titers in the serum, spleen and brain than wild-type (WT) mice and expanded numbers of newly formed T1 B cells. Remarkably, and unlike fully B cell-deficient μMT mice, sustained protective immunity could be induced in the mature B-cell deficient BAFFR^-/-^ mice by either passive transfer of immune sera or by vaccination with the WNV envelope protein (WNV E) conjugated to an anti-CD180 Ab. Thus, newly formed B cells in BAFFR^-/-^ mice can contribute to the development of protective immunity.

## Results

### BAFFR^-/-^ mice succumb to WNV infection

**Fig 1:**
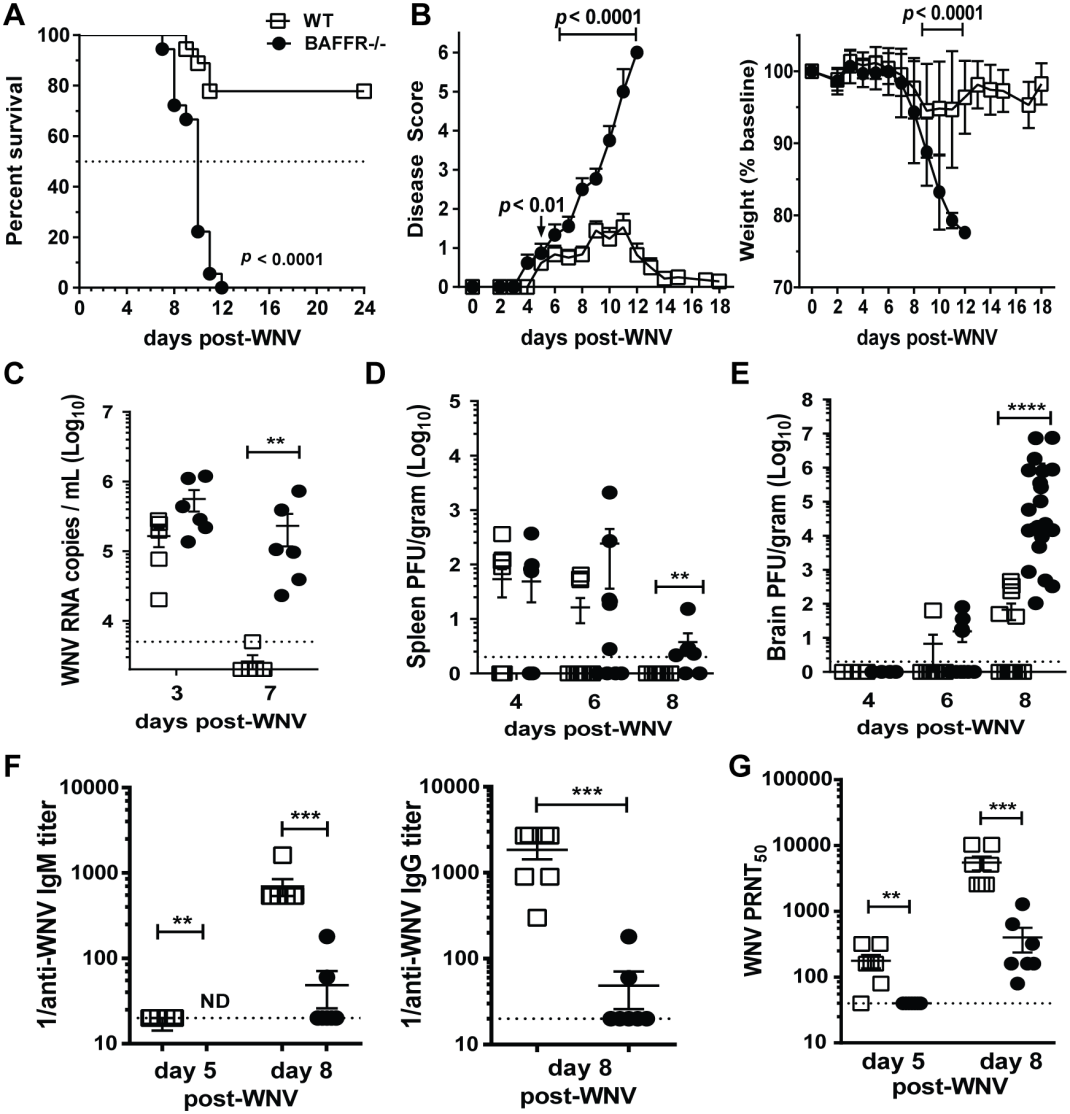
BAFFR^-/-^ mice have increased mortality, increased viral titers, and reduced and delayed WNV-specific and neutralizing Ab responses after WNV infection. Adult C57BL/6 WT mice (white squares) and BAFFR^-/-^ mice (black circles) were inoculated s.c. with 10^2^ PFU of WNV (strain Texas 2002) and monitored daily for survival (*A*) and clinical signs of disease (*B*); Sera (*C*) and tissues (*D* and *E*) were harvested at the indicated day post-WNV infection; and virus-specific Abs (*F*) or nAbs responses (*G*) in sera were measured at the indicated time points post-infection. *A*, Statistics were performed using a log-rank test for significance comparing the percentage of surviving WT mice to that of BAFFR^-/-^ mice. *B*, Clinical course of disease (*left panel*). Error bars represent variance of clinical scores. Weights (*right panel*) are reported as % of the mouse weight before infection. Significance was determined with the multiple *t* test Holm-Sidak method, significant *p* values are shown. *A* and *B*, graphs summarize data from four independent experiments (N = 18). Viral titers in sera were quantified using real-time qPCR (*C*), and were determined in tissues using a standard plaque assay (*D*, and *E*). Graphs show PFU of virus per gram of tissue or copies of viral RNA per ml serum. (*C-E*), Graphs summarize data from two independent experiments; C, N = 6; *D* and *E*, N = 6–15. The dotted lines represent the limit of detection. Statistics were determined by two-tailed Mann-Whitney *t* test, ** *p*<0.01, *** *p*<0.001. (*F*) Anti-WNV E -IgM (*left panel*) and -IgG (*right panel*) were measured by ELISA, each symbol shows relative titers from an individual mouse. ND, not detected. (*G*) nAb titers were determined by a PRNT assay, with 50% virus inhibitory capacity. In *F* and *G*, data show one representative experiment (N = 7) out of more than three independent infections with at least four mice per group per time point. The dotted lines represent the limit of detection. Statistics were determined by two-tailed Mann-Whitney *t* test, ** *p*<0.01, *** *p*<0.001.

A deficiency of BAFFR can affect mice differently depending on the type of viral infection [12,13,14]. We assessed the susceptibility of BAFFR^-/-^ mice to WNV using a pathogenic North American isolate from Texas (WNV-TX) [28]. BAFFR^-/-^ mice, in contrast to WT mice, were highly susceptible after a sub-lethal s.c. inoculation of WNV (Fig 1A and 1B). 100% of BAFFR^-/-^ mice died (median survival time, 8–10 days) compared to 22% of WT mice (*p* < 0.0001) (Fig 1A). Whereas symptoms began to resolve in WT mice by 10–12 days post-infection (p.i.), paralysis became evident in many BAFFR^-/-^ mice by day 7 p.i. (Fig 1B, *left panel*). WT mice that survived infection began to recover from weight loss by around day 8 p.i., yet BAFFR^-/-^ mice had lower weights than WT mice by day 9 p.i. (Fig 1B, *right panel*). The course of the disease in BAFFR^-/-^ mice was similar to that in fully B cell-deficient μMT mice [22,23]. Thus, BAFFR and mature B cells are essential for protection against pathogenic WNV infection.

### Decreased virus clearance and increased viral burdens in the brains of BAFFR^-/-^ mice after WNV infection

To define the role of BAFFR and mature B cells in controlling WNV *in vivo*, we infected WT and BAFFR^-/-^ mice with 10^2^ plaque forming units (PFU) of WNV-TX and monitored viral burden within peripheral tissues and the CNS over time. We measured viral RNA in the sera of infected WT and BAFFR^-/-^ mice by quantitative polymerase chain reaction (qPCR). Compared to WT mice, BAFFR^-/-^ mice exhibited no significant increase in viremia at early time points (Fig 1C). However, at day 7 p.i. when the virus was cleared in the sera of WT mice, BAFFR^-/-^ mice had high levels of viral mRNA (Fig 1C). There were no significant differences in virus levels within the kidneys of infected BAFFR^-/-^ vs. WT mice; and the kinetics and peak of virus levels (day 4) in the spleen were similar between infected WT and BAFFR^-/-^ mice (Fig 1D). However, by day 8 p.i., in contrast to WT mice, BAFFR^-/-^ mice failed to clear virus from the spleen (Fig 1D). As seen in previous studies [29], some WT mice had detectable WNV loads in the brain by day 6 post-infection that increased by day 8 p.i. (Fig 1E). The kinetics of WNV infection in the brain was similar in BAFFR^-/-^ and WT mice, except that BAFFR^-/-^ mice had substantially increased viral loads at day 8 p.i. (Fig 1E). These results suggest that BAFFR and mature B cells are not essential to control virus replication at early stages of WNV infection, but they are critical for peripheral organ clearance and preventing virus infection of the CNS.

### Defective WNV-specific B cells responses in BAFFR^-/-^ mice

We examined if the higher susceptibility to WNV and the inability to control virus infection of the brain in BAFFR^-/-^ mice was due to defective WNV-specific IgM and IgG responses. WNV E-specific IgM was detectable albeit at low levels in WT mice at day 5 p.i. and then increased by day 8 (Fig 1F, *left panel*). In contrast, WNV E-specific IgM was undetectable in the sera of BAFFR^-/-^ mice at day 5 p.i., and the IgM levels at day 8 p.i. were >25-fold lower than in WT mice (Fig 1F, *left panel*). Similarly, WNV E-specific IgG levels were lower (~135-fold) in the sera of BAFFR^-/-^ versus WT mice at day 8 p.i. (Fig 1F, *right panel*).

We next assessed WNV-specific nAb responses using a plaque reduction neutralization test (PRNT) and found that nAb titers in sera from BAFFR^-/-^ mice were ~4-fold lower than WT mice at day 5 p.i. (Fig 1G). As expected nAb titers in the sera of WT mice increased markedly between day 5 and day 8 p.i. (Fig 1G). WNV-specific nAbs were lower (~32-fold) in BAFFR^-/-^ versus WT mice at day 8 p.i. (Fig 1G). However, in spite of a deficiency in formation of mature B cells, nAb titers in BAFFR^-/-^ mice rose 4-fold between day 5 and day 8 (Fig 1G).

**Fig 2:**
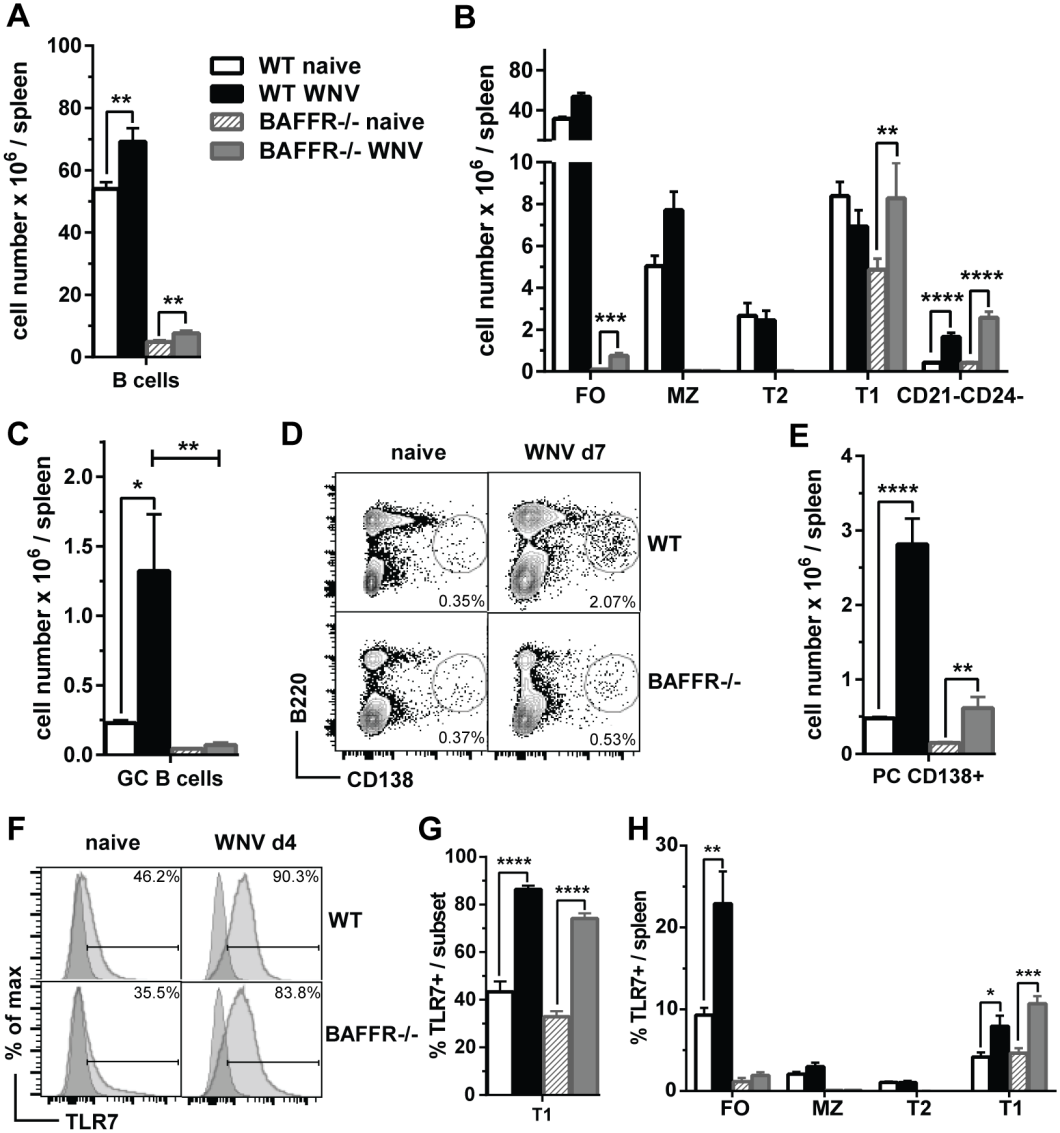
BAFFR^-/-^ mice are defective in FO, MZ and T2 B cells and after WNV-TX infection do not up-regulate GC B cells and have reduced PCs. Spleens from WT and BAFFR^-/-^ naïve mice or mice infected for 7 days (*A*-*E*) or 4 days (*F* and *G*) with 10^2^ PFU of WNV-TX were harvested and cell populations were determined by flow cytometry. Total splenocytes were stained with surface markers for total B cells (*A*), B cell subsets (*B*), GC B cells (*C*) and PCs (*D* and *E*). The gating strategy used to identify splenic B cell subsets (*A* and *B*) is shown in S1 Fig). GC B cells (*C*) were identified as GL7^+^CD38^-^ B cells (gated on B220^+^ cells). PCs (*D* and *E*) are quantified as frequencies of B220^lo^CD138^+^ cells in representative dot plots from three independent experiments (*D*) or as total cell numbers (*E*). In *F* and *G* frequencies of TLR7^+^ T1 B cells quantified by flow cytometry and shown as representative histograms (*F*) or bar graphs (*G*) from two independent experiments. *H* shows the frequencies of TLR7^+^ B cell subsets in the spleens of WT and BAFFR^-/-^ mice before and 4 days after WNV infection. In *A*, B, *C*, *E*, *G* and *H* graphs show means ± SEM of absolute numbers and summarize data from three (*A*, *B*, *C* and *E*) or two (*G* and *H*) independent experiments (N = 9 or N = 6, respectively) In *A*, B, *E*, *G* and *H* statistics comparing naïve vs. infected mice were determined by two-tailed Student’s *t* test; in *C* statistics was performed by one-way ANOVA corrected with Holm-Sidak method for multiple comparisons; * *p*<0.05, ** *p*<0.01, *** *p*<0.001, **** *p*<0.0001.

Since BAFFR^-/-^ mice had defective Ab responses but still produced some WNV-specific nAbs, we examined whether there were qualitative changes in BAFFR^-/-^ B cell subsets in response to WNV challenge. As previously reported, the number of splenic B cells was reduced in BAFFR^-/-^ naïve mice compared to WT mice (Fig 2A); seven days after WNV infection, B cells expanded in the spleen of both BAFFR^-/-^ mice and WT mice, although numbers were lower in BAFFR^-/-^ mice (Fig 2A). Consistent with previous studies [9,10], naïve BAFFR^-/-^ mice lacked MZ and T2 cells and had almost no mature FO B cells (Fig 2B, gating strategy in S1A Fig). However, BAFFR^-/-^ mice had relatively normal levels of newly formed T1 B cells. As expected, after WNV infection, splenic FO and MZ B cells expanded in WT mice. Additionally, despite their early developmental stage, T1 B cells from BAFFR^-/-^ mice also increased almost two-fold after WNV infection (Fig 2B).

To test whether T1 B cells expanded by WNV infection in BAFFR^-/-^ mice retained an “immature” phenotype, we examined expression of CD93 (AA4.1), a marker of newly formed B cells [30]. T1 B cells from both WT and BAFFR^-/-^ mice either before or after infection expressed similarly high levels of CD93 (S1B Fig, *upper panel*), indicating that they retained their immature phenotype. In addition, the small number of FO-like B cells, representing about 5% of the total B cells, also was increased in BAFFR^-/-^ mice (Fig 2B). Unlike the CD93^-^ FO B cells in WT mice, most of these B cells classified as FO B cells based on their expression of CD21 and CD24, in infected BAFFR^-/-^ mice also expressed CD93 (S1B Fig, *lower panel*). These data suggest that the small number of FO-like B cells in infected BAFFR^-/-^ mice were developmentally immature compared to FO B cells from infected WT mice. Furthermore, and in contrast to WT mice, BAFFR^-/-^ mice did not develop CD38^-^GL7^+^ germinal center (GC) B cells 7 days after WNV infection, consistent with their virtual absence of mature FO B cells (Fig 2C). In addition, naïve BAFFR^-/-^ mice had substantially fewer splenic (S1D Fig) and peritoneal B1 B cells (S1E Fig *left panel*) compared to WT mice. The numbers of splenic B1 and peritoneal cavity B1a and B1b cells did not change in BAFFR^-/-^ mice after WNV infection (S1D and S1E Fig *left panel*) suggesting they do not contribute to WNV immunity. We conclude that, after WNV infection of BAFFR^-/-^ mice, the immature splenic B cells were the major B cell subset that expanded significantly.

We also measured the levels of splenic B220^lo^ CD138^+^ PCs in WT and BAFFR^-/-^ mice 7 days after WNV infection. Infected BAFFR^-/-^ mice had fewer PCs than WT mice (Fig 2D and 2E). Nonetheless, splenic PC numbers were increased after WNV infection in both WT and BAFFR^-/-^ mice, although to a lesser extent in BAFFR^-/-^ mice. A B220^+^CD21^-^CD24^-^ B cell subset also was increased significantly in both WT and BAFFR^-/-^ mice after WNV infection (Fig 2B). These B220^+^ cells may be precursors of plasmablasts or PCs. Indeed, a fraction of these cells were CD138^+^ (S1C Fig) and as B cells mature into terminal PCs, they cease to express CD21 and CD24 [31,32]. Further studies are required to define this B cell population. In addition, we did not detect any increase in PCs in the peritoneal exudate from WNV-infected WT or BAFFR^-/-^ mice (S1E Fig *right panel)*.

Type I interferon (IFN) can upregulate the expression of Toll-like receptor 7 (TLR7) in B cells including immature B cells enabling them to be more responsive to viral RNA [33,34]. Thus, we tested whether T1 B cells from BAFFR^-/-^ mice upregulated TLR7 expression after WNV infection. In naïve WT mice T2 and MZ B cells expressed the highest levels of TLR7, while T1 and FO B cells upregulated TLR7 expression upon WNV infection (Fig 2F and 2G). T1 B cells from infected BAFFR^-/-^ mice substantially increased their TLR7 expression to levels observed in infected WT mice (Fig 2F and 2G). Thus, while in WT mice both FO and T1 B cells upregulated TLR7 upon infection, in BAFFR^-/-^ mice T1 B cells were the major B cell subset responding to WNV that increased TLR7 expression (Fig 2H). In summary, BAFFR^-/-^ B cells responded to WNV challenge and could develop into PCs, but did not develop into GC B cells. After WNV infection, T1 B cells upregulated TLR7 and expanded significantly.

### After WNV infection BAFFR^-/-^ mice have lower T cell responses and lower numbers of responding DCs

**Fig 3:**
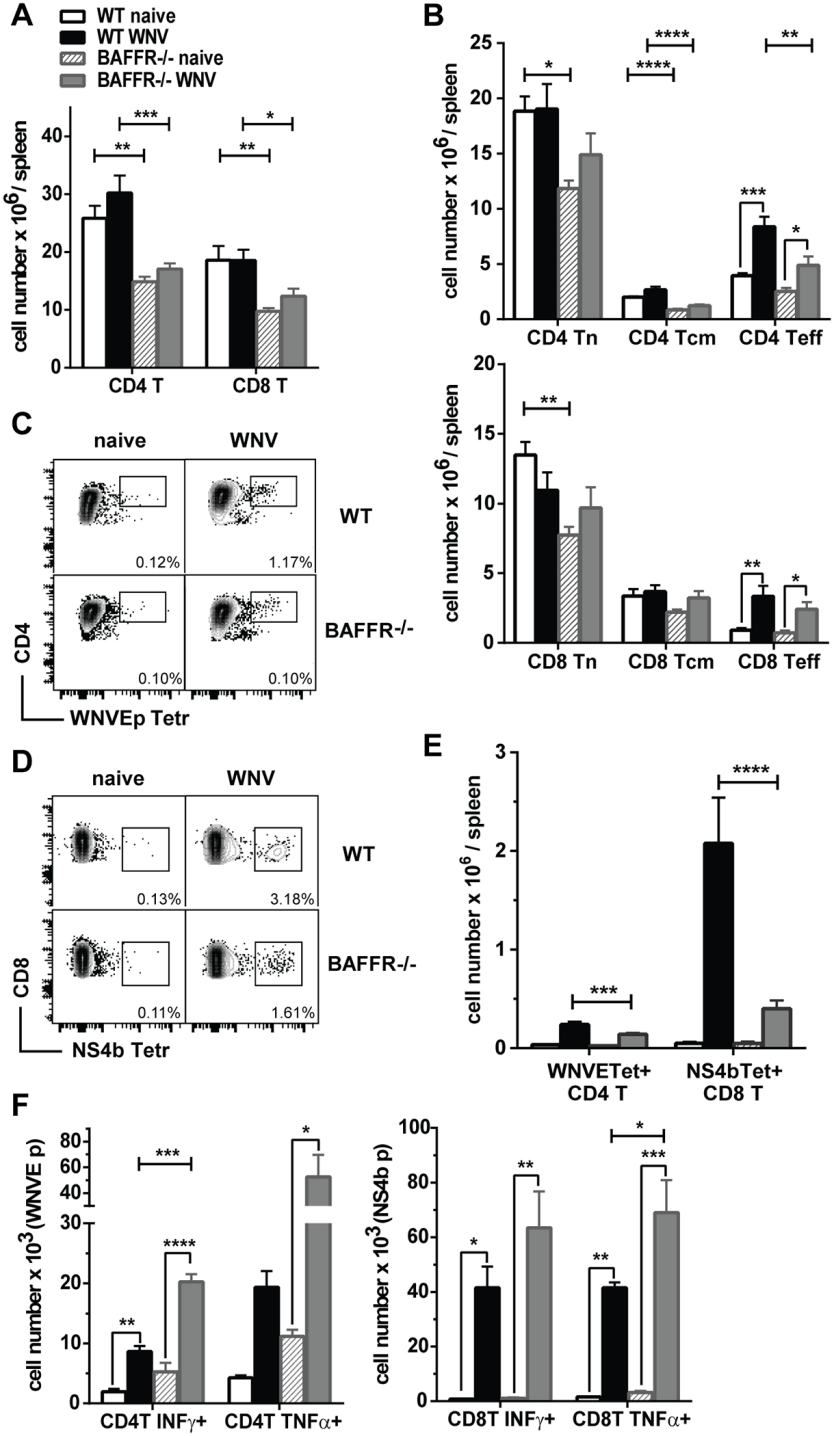
BAFFR^-/-^ mice have reduced T cell responses early after WNV infection but their CD4^+^ and CD8^+^ T cells respond normally to WNV-specific peptides *in vitro*. *A-F*, Spleens from WT and BAFFR^-/-^ naïve mice or from mice 7 days after s.c. WNV infection were harvested, and cell populations were determined by flow cytometry. In *A*, absolute numbers of CD4^**+**^ and CD8^**+**^ T cells. In *B*, CD4^**+**^ T and CD8^**+**^ T cells subpopulations were determined as described in the ***Methods*** and are defined as: naïve (Tn), central memory (Tcm) and effector (Teff) T cells. WNVE-tetramer+ CD4^**+**^ T cells and NS4b-tetramer+ CD8^**+**^ T cells are shown as frequencies in representative dot plots (*C* and *D*) and as absolute cell numbers (*E*). In *A*, *B* and *E*, graphs summarize data from three independent experiments (N = 9), except for NS4b Tetramer^+^ CD8^**+**^ T cells where the graph summarizes data from two independent experiments (N = 6). In *F*, naïve or WNV infected splenocytes were stimulated with 1 μM WNV E_641-655_ peptide (*left panel*) or 1 μM NS4B H-2D peptide (*right panel*) for 6 h *in vitro*. Reported are cell numbers of IFNγ^+^ or TNFα^+^ CD44^+^ CD4^**+**^ T cells (*left panel*) or CD8^**+**^ T cells (*right panel*) from one representative out of two independent experiments with three mice per group. In *A*, *B*, *E* and F bar graphs show means ± SEM; * *p*<0.05, ** *p*<0.01, *** *p*<0.001, as determined by one-way ANOVA corrected with Holm-Sidak method for multiple comparisons.

We assessed whether other cells involved in innate and adaptive immunity were intact in naïve and WNV-infected BAFFR^-/-^ mice. CD4^+^ and CD8^+^ T cells are essential to control viral dissemination to the CNS at later stages after WNV infection [20,21]. Both CD4^+^ and CD8^+^ T cell numbers were reduced in the spleens of naïve BAFFR^-/-^ mice compared to naïve WT mice (Fig 3A). These T cell subset reductions were maintained, but less pronounced after WNV challenge. We further subdivided the splenic CD4^+^ and CD8^+^ T cell subsets from uninfected and WNV-infected mice into naïve (Tn), central memory (Tcm) and effector (Teff) T cell subpopulations (Fig 3B, and see Materials and methods). The numbers of CD4^+^ and CD8^+^ Teff cells were selectively increased in both WT and BAFFR^-/-^ mice (Fig 3B).

We also examined Ag-specific T cell responses using either a MHC class II tetramer specific for an immunodominant WNV E peptide bound by CD4^+^ T cells (Fig 3C) or a MHC class I tetramer specific for an immunodominant NS4B peptide bound by CD8^+^ T cells (Fig 3D). Although both WT and BAFFR^-/-^ mice had increased numbers of splenic WNV-specific CD4^+^ and CD8^+^ T cells at day 7 p.i., the numbers were lower in BAFFR^-/-^ mice compared to WT mice (Fig 3E).

To assess whether WNV-specific CD4^+^ and CD8^+^ T cells in WNV-infected BAFFR^-/-^ mice had reduced poly-functionality (Fig 3E), we evaluated cytokine production upon re-stimulation with a WNV E peptide (CD4^+^ T cells) or NS4B peptide (CD8^+^ T cells). WNV peptide re-stimulation induced similar levels of IFNγ- and TNFα- producing CD44^+^ CD4^+^ and CD44^+^ CD8^+^ T cells from either WT or BAFFR^-/-^ mice (Fig 3F). If anything, there were more IFNγ- and TNFα-secreting CD44^+^ CD4^+^ T cells in splenic cultures from BAFFR^-/-^ than WT mice. Thus, the reduction in WNV-specific T cell levels in BAFFR^-/-^ mice is not due to an intrinsic defect in the ability of T cells to respond to the virus in the absence of BAFFR.

**Fig 4:**
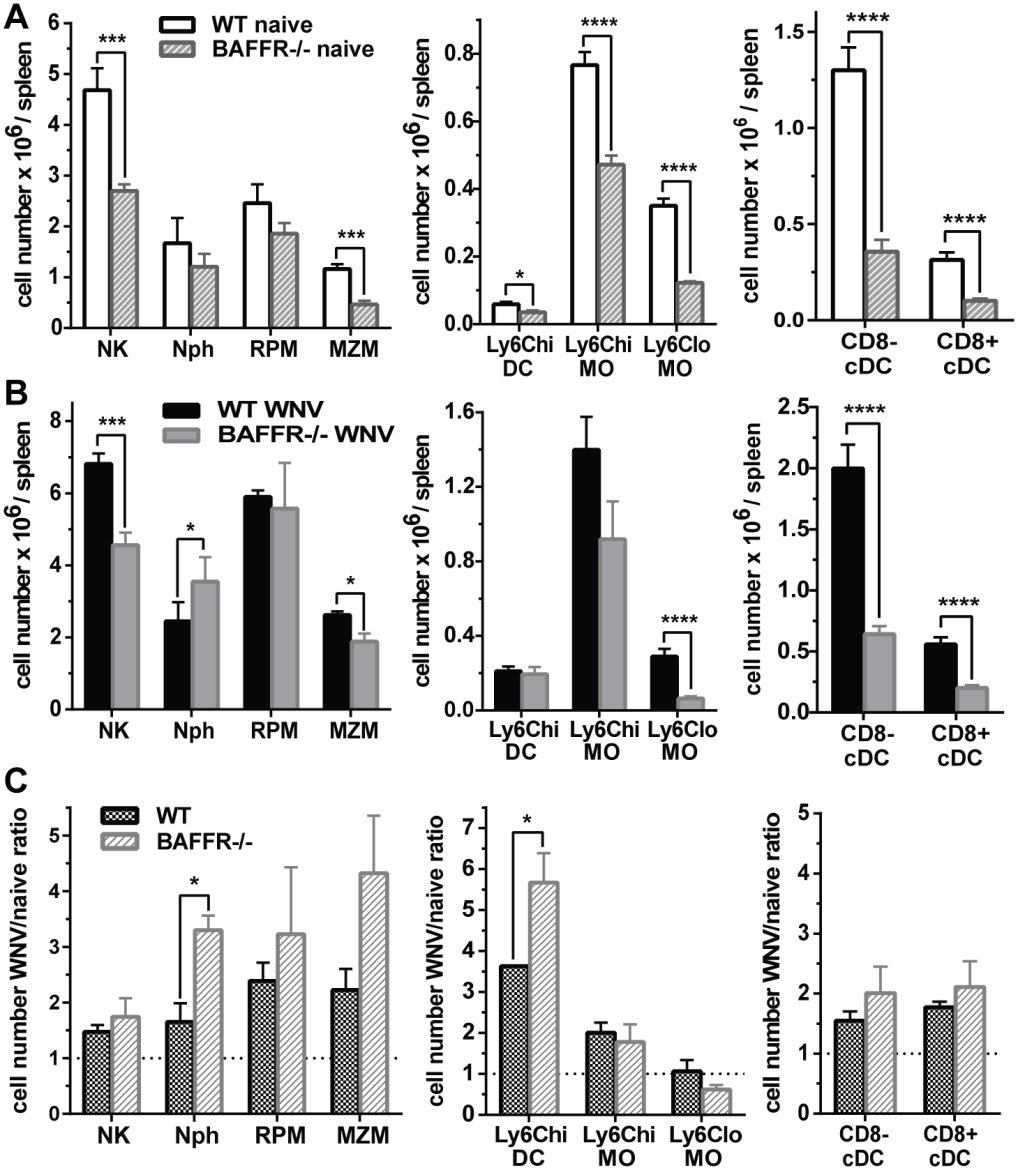
Myeloid cell and DC subsets are reduced in naïve and WNV-infected BAFFR^-/-^ mice. Spleens from WT and BAFFR^-/-^ naïve mice (*A*) or mice infected for 7 days with WNV (*B*) were harvested and cell populations were determined by flow cytometry. *A* and *B*, NK cells (NK), Neutrophils (Nph), Red Pulp Macrophages (RPM); Marginal Zone Macrophages (MZM), Ly6C^hi^ inflammatory DCs (Ly6C^hi^ DC); Ly6C^hi^ MOs (Ly6C^hi^ MO), Ly6C^lo^MOs (Ly6C^lo^ MO), CD8^-^ cDCs (CD8^-^ cDC), CD8^+^ cDCs (CD8^+^ cDC). In *A* and *B* graphs show absolute numbers. *C*, Ratio of absolute cell numbers of WNV infected (*B*) vs. naïve (*A*) WT and BAFFR^-/-^ mice. Data are represented as means ± SEM from three independent experiments using three mice/group each (N = 9), except for MZM and RPM which are from two independent experiments (N = 6). Statistics were determined by two-tailed Student’s *t* test, * *p*<0.05, ** *p*<0.01, *** *p*<0.001, **** *p*<0.0001.

In agreement with a recent study that reported a reduction in CD169^+^ MZMs in BAFFR^-/-^ mice [14], we detected reduced splenic MZMs numbers in BAFFR^-/-^ mice either prior to or at 7 days p.i., as well as reduced numbers of CD8^+^ and CD8^-^ cDCs, Ly6C^lo^ MOs, and NK cells (Fig 4A and 4B, gating strategy in S2 Fig). However, inflammatory Ly6C^hi^ MOs and RPM, which can promote pathogenesis in WNV-induced encephalitis [35,36,37,38], were not reduced, and Nphs levels were greater in infected BAFFR^-/-^ than WT mice (Fig 4B). Given these changes, we calculated the relative increase in each population after WNV infection (Fig 4C). Overall, myeloid populations were expanded equivalently in WT and BAFFR^-/-^ mice after WNV infection, with a somewhat greater expansion of Nphs and Ly6C^hi^ DCs in BAFFR^-/-^ than WT mice. Thus, except for the lack of mature B cells, innate and adaptive immune responses to WNV, although somewhat reduced, were not significantly disrupted in BAFFR^-/-^ mice.

### Immune serum from WNV-infected WT mice induces long term protection in BAFFR^-/-^ but not μMT mice

**Fig 5:**
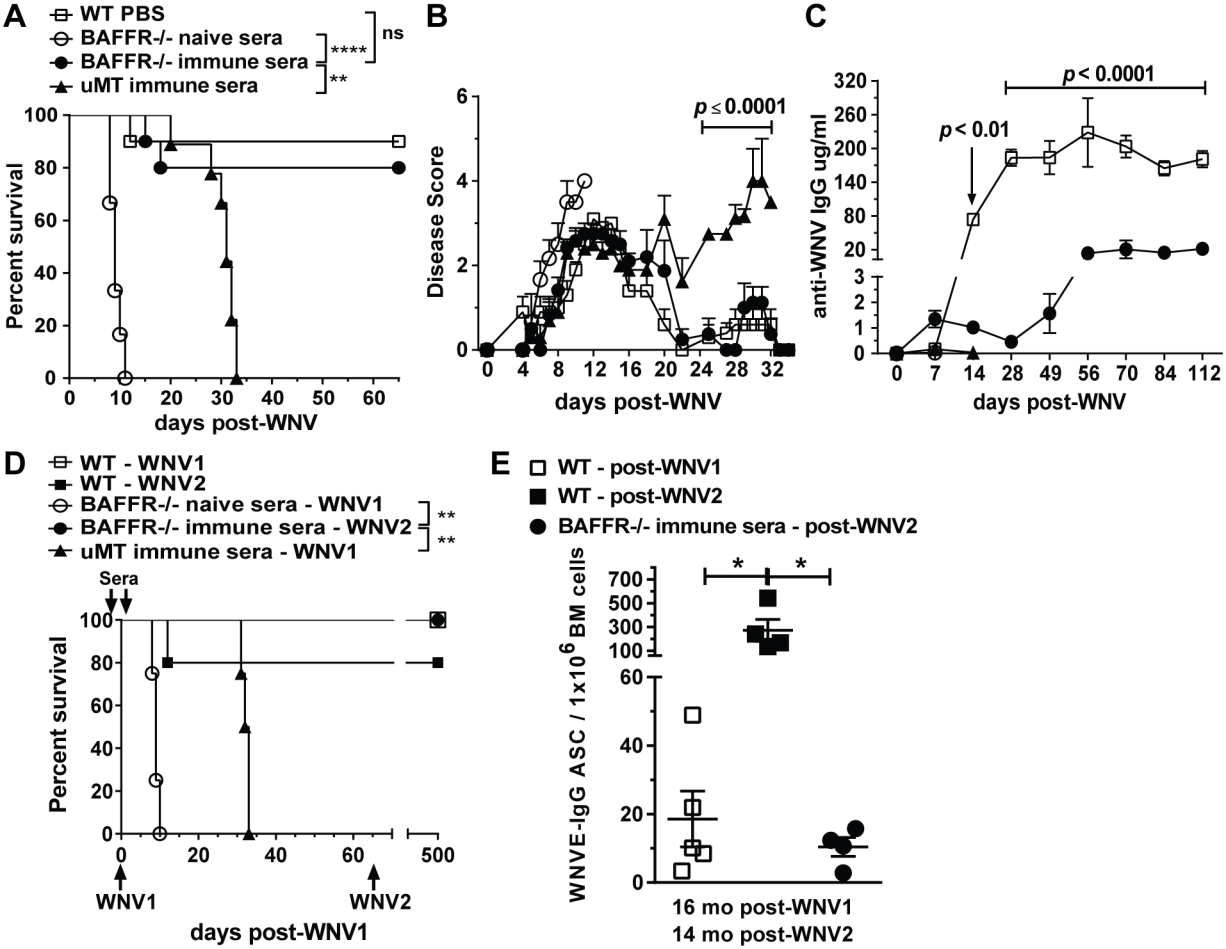
Transfer of WNV immune sera induces long term protection against WNV in BAFFR^-/-^ but not μMT mice. *A*-*E*, Heat-inactivated sera obtained from WT mice infected for 5 to 14 days with WNV were inoculated into BAFFR^-/-^ and μMT mice 1 day prior and 1 day after WNV infection (See Methods). In *D* and *E*, 65 days after the first WNV infection (WNV1) mice were re-challenged with 10^2^ PFU of WNV-TX (WNV2) and 14 months later bone marrows from surviving mice were harvested to measure long-lived WNV E-specific Ab secreting cells (ASCs) by ELISPOT (*E*). Mice were monitored for survival (*A* and *D*) and clinical signs of disease (*B*). *C*, WNV E-specific IgG in sera from WT, BAFFR^-/-^ and μMT mice at the indicated time points after WNV infection was detected by ELISA. *A*-*C*, graphs summarize data from two independent experiments; N = 10 for WT mice given PBS, BAFFR^-/-^ mice inoculated with WT immune sera and μMT mice inoculated with WT immune sera, and N = 6 for BAFFR^-/-^ mice inoculated with WT naïve sera. In *D*, data are from one experiment; N = 5 for WT mice infected once (WNV1) or two times (WNV2); N = 4 for BAFFR^-/-^ mice inoculated with WT naïve sera, BAFFR^-/-^ mice inoculated with WT immune sera and infected twice (WNV2) and μMT mice inoculated with WT immune sera and infected once (WNV1). In *A* and *D* statistics were performed using a log-rank test for significance, **, *p*<0.01; ****, *p*<0.0001; n.s., not significant. The multiple *t* test Holm-Sidak method was used to compare in *B*, BAFFR^-/-^ mice that received immune sera and μMT mice that received immune sera; and in *C*, WT mice that received PBS and BAFFR^-/-^ mice that received immune sera. In *E* data are represented as means ± SEM from one experiment and statistics were determined by one-way ANOVA corrected with Holm-Sidak multiple comparison post-test, * *p*<0.05.

B cell-deficient μMT mice can be protected temporarily against WNV-induced death by passive transfer of immune sera from previously infected WT mice [22,23]. To assess if this was also the case for BAFFR^-/-^ mice that lack mature B cells, we passively administered into BAFFR^-/-^ mice either immune sera collected from WT mice that had cleared virus or normal control sera (Fig 5A). Heat-inactivated sera were administered 1 day prior to and after infection with WNV as described for μMT mice [22]. In contrast to the BAFFR^-/-^ mice given control sera, which all succumbed to infection, BAFFR^-/-^ mice receiving immune sera were protected from WNV infection, as were control μMT mice receiving immune sera (Fig 5A). By about three weeks post-infection almost all BAFFR^-/-^ mice showed no clinical signs of disease; however, infected μMT mice showed some paresis of the lower limbs (Fig 5B). As expected [39], at 30 days after infection μMT mice that had received immune sera continued to show signs of disease progression and were dead by day 33 (Fig 5A and 5B). In contrast, BAFFR^-/-^ mice that had received immune sera all survived for several months (Fig 5A).

The results above suggested that BAFFR^-/-^ mice might be able to develop the humoral immune responses necessary to overcome a second infection. Thus, we assessed whether surviving BAFFR^-/-^ mice produced WNV-specific IgGs at later time points after WNV infection and at higher levels than achieved after passive transfer (Fig 5C). BAFFR^-/-^ mice given immune sera developed higher levels of WNV E-specific IgGs than detected early 7–28 days after infection (0.5–1.5 μg/ml), which reached a plateau of about 20 μg/mL by 56 days post-infection. However, the levels of WNV-specific IgGs produced by BAFFR^-/-^ mice were lower than in infected WT mice. BAFFR^-/-^ mice that received immune sera were able to overcome a challenge with WNV 65 days after the first infection and survived for more than a year (Fig 5D). In addition, 14 months after the re-challenge BAFFR^-/-^ mice that received immune sera and were infected twice had long-lived WNV E-specific Ab secreting cells (ASCs) at levels comparable to levels in WT mice infected once but less than in WT mice infected twice (Fig 5E). Thus, although BAFFR^-/-^ mice lacked mature B cells, they nonetheless produced somewhat delayed yet long-lived WNV-specific humoral responses that enabled long-term survival after challenge.

To understand what might account for the differential survival of BAFFR^-/-^ and μMT mice given immune sera, we examined whether in addition to B cells there were differences in other cell populations (S3A Fig). Naive μMT mice had lower numbers of T cells, NK cells, RPM and DCs compared to BAFFR^-/-^ mice. However, 7 days after WNV infection BAFFR^-/-^ and μMT mice had no significant differences in these splenic cell populations (S3B Fig). Thus, the major difference between BAFFR^-/-^ and μMT mice is that BAFFR^-/-^ mice have newly formed B cells, whereas μMT mice do not.

### Vaccination with WNV E-anti-CD180 conjugate protects BAFFR^-/-^ mice from WNV infection

The ability of BAFFR^-/-^ mice to be protected by immune sera and survive for a long time after challenge suggested that vaccination might protect them from lethal WNV infection. To test this concept, we created a vaccine based on our finding that conjugates comprised of Ags coupled to anti-CD180 Abs, when inoculated into mice rapidly expand T1 B cells and also induce potent and long-lasting Ag-specific IgG Ab responses even in BAFFR^-/-^ mice [27]. We conjugated purified, recombinant WNV E protein to a rat anti-mouse CD180 mAb, inoculated graded doses (10 to 50 μg) of WNV E-αCD180 into WT mice, and selected a dose (20 μg) that efficiently induced WNV E-specific IgG and nAbs.

**Fig 6:**
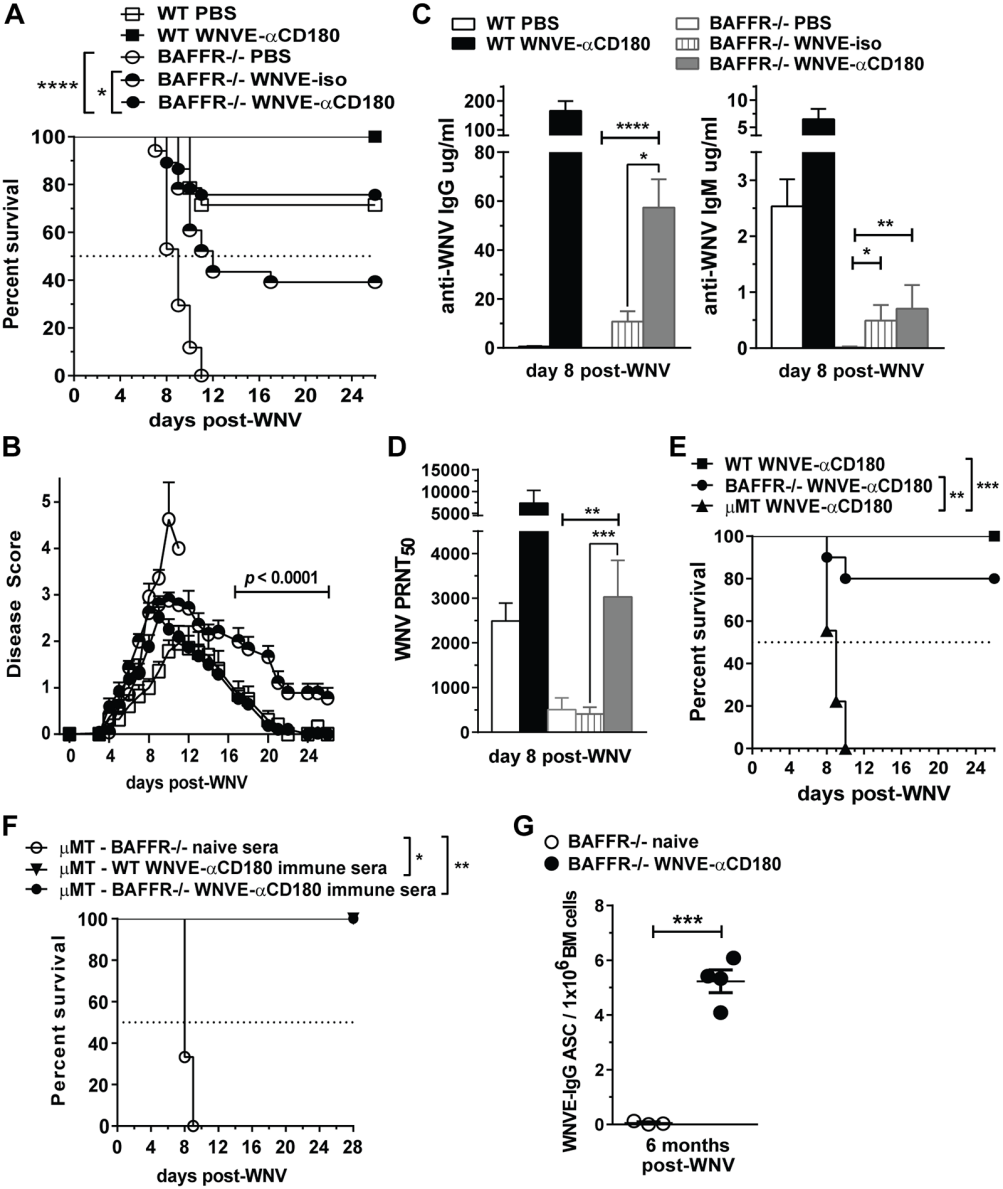
Vaccination with a WNV E-αCD180 conjugate protects BAFFR^-/-^ mice from WNV infection and enhances WNV E-specific Abs responses. *A*-*D*, WT and BAFFR^-/-^ mice were inoculated i.v. with PBS, 20 μg/mouse of WNV E- anti-CD180 protein conjugate (WNVE-αCD180) or WNV E-ratIgG2a (WNVE-iso) 30 days prior to s.c. infection with WNV (10^2^ PFU). Mice were monitored daily for survival (*A*) and clinical signs of disease (*B*). *A*, statistics were performed using a log-rank test for significance. *B*, the multiple *t* test Holm-Sidak method was used to determine significance. *A* and *B*, graphs summarize data from >4 independent experiments, N = 14 for WT mice given PBS, N = 6 for WT mice immunized with WNV E-αCD180, N = 17 for BAFFR^-/-^ mice given PBS, N = 23 for BAFFR^-/-^ mice immunized with WNV E-iso, N = 37 for BAFFR^-/-^ mice immunized with WNV E-αCD180. Anti-WNV E IgM and IgG measured by ELISA (*C*) and anti-WNV nAbs measured by PRNT (*D*) in sera harvested 8 days after WNV infection. *C* and *D*, graphs summarize data from three independent experiments, N = 9 for WT mice given PBS, N = 6 for WT mice immunized with WNV E-αCD180, N = 9 for BAFFR^-/-^ mice given PBS, N = 12 for BAFFR^-/-^ mice immunized with WNV E-iso, N = 12 for BAFFR^-/-^ mice immunized with WNV E-αCD180. In *C* and *D* statistics were determined by Kruskal-Wallis corrected with Dunn’s multiple comparisons post-test. *E*, WT, BAFFR^-/-^ and μMT mice were inoculated i.v. with 20 μg/mouse WNV E-αCD180 30 days prior s.c. infection with WNV. Survival data are from two independent experiments (N = 10). Statistics were determined using the log-rank test for significance. *F*, Heat-inactivated sera obtained from naïve BAFFR^-/-^ mice (open circles), WT mice vaccinated with WNVE-αCD180 (triangles) or from BAFFR^-/-^ mice vaccinated with WNVE-αCD180 (closed circles) and challenged with WNV (sera obtained between day5 and day 28 post-infection), were inoculated into μMT mice 1 day prior and 1 day after WNV infection (See Methods) and mice were monitored for survival. Survival data are from one experiment (mice receiving either naïve BAFFR^-/-^ sera or immune WT sera: N = 3; mice receiving immune BAFFR^-/-^ sera: N = 6) and statistics were performed using a log rank test for significance. *G*, BAFFR^-/-^ mice were vaccinated with WNVE-αCD180 and infected with WNV (as in *A*), 6 months later bone marrows were harvested to measure long-lived WNV E-specific ASCs by ELISPOT. In *G*, data are represented as means ± SEM from one experiment and statistics were determined by two-tailed Student’s *t* test. For all statistical analyses significant *p* values are indicated as follows: * *p*<0.05, ** *p*<0.01, *** *p*<0.001, **** *p*<0.0001.

Immunization of BAFFR^-/-^ mice with WNV E-αCD180 induced E protein-specific IgG Abs, but the responses were slower and lower than in WT mice (S4A Fig). Administration of one dose of WNV E-αCD180 to BAFFR^-/-^ mice 30 days prior to WNV infection protected 76% of BAFFR^-/-^ mice in contrast to the 100% mortality in BAFFR^-/-^ mice immunized with PBS (Fig 6A). Thus, WNV E-αCD180 immunization induced protection in mature B cell-deficient BAFFR^-/-^ mice. As expected, WNV E-αCD180 immunization also improved survival of WT mice, with 100% survival after vaccination (Fig 6A). In contrast, when we immunized BAFFR^-/-^ mice with soluble WNV E protein (WNV Ep) only or the anti-CD180 mAb only, all the mice succumbed to infection by day 13 (S4B Fig). Thus, as with protein Ags [27], WNV E protein Ag and anti-CD180 together were required to induce protection of BAFFR^-/-^ mice against WNV infection. As an additional control, we immunized BAFFR^-/-^ mice with a non-binding rat IgG2a isotype mAb conjugated with WNV E protein (WNV E-iso). In this case we observed some protection, as 39% of BAFFR^-/-^ mice immunized with WNV E-iso survived (Fig 6A), but this level that was lower than those immunized with WNV E-αCD180. Furthermore, while BAFFR^-/-^ mice immunized with WNV E-αCD180 lacked signs of clinical disease by day 20 post infection, BAFFR^-/-^ mice immunized with WNV E-iso that survived infection displayed some mild paresis of the lower limbs even up to 26 days post-challenge (Fig 6B).

Next, we examined if WNV E-αCD180-induced protection in BAFFR^-/-^ mice was the result of an increase in WNV-specific humoral immunity. Indeed, BAFFR^-/-^ mice immunized with WNV E-αCD180 had a substantial increase in WNV E-specific IgG 8 days post-challenge compared to mice given PBS (Fig 6C). The levels of WNV E-specific IgG were higher at day 8 post-infection in both WT and BAFFR^-/-^ mice that were immunized and infected compared to uninfected mice evaluated 28 days after WNV E-αCD180 immunization (9.7-fold for WT mice, *p* = 0.002; 6.3-fold for BAFFR^-/-^ mice, *p* = 0.0003, Fig 6C, S4A Fig). Even though immunization with WNV E-iso induced some WNV E-specific IgG vs PBS in BAFFR^-/-^ mice, the levels were lower than in mice immunized with WNV E-αCD180 (Fig 6C, *left panel*). In contrast, both WNV E-iso and WNV E-αCD180 upregulated WNV E-specific IgM to a similar extent compared to PBS-treated BAFFR^-/-^ controls, but the levels were lower than those in PBS-treated WT mice (Fig 6C, *right panel*). Indeed, WNV E-αCD180 immunization induced more anti-WNV IgG versus anti-WNV IgM in both WT and BAFFR^-/-^ mice (Fig 6C). Furthermore, only immunization with WNV E-αCD180 induced a substantial increase in nAb in BAFFR^-/-^ mice, while WNV E-iso immunization did not (Fig 6D). Although the nAb titers in sera from WNV E-αCD180 immunized BAFFR^-/-^ mice were lower than WNV E-αCD180 immunized WT mice, they were comparable to the level of nAbs induced in WT PBS control mice (Fig 6D). These results suggest that the greater protection of BAFFR^-/-^ mice provided by WNV E-αCD180 compared to WNV E-iso immunization, likely is due to WNV E Ag targeting to B cells via CD180, which promoted class switching and production of nAbs. To determine whether B cells were required for WNV E-αCD180 vaccine to be effective we immunized μMT mice with WNV E-αCD180 30 days prior WNV inoculation. In contrast to WNV E-αCD180 immunized BAFFR^-/-^ mice, all μMT mice succumbed to infection (Fig 6E). Thus, the immature B cells present in BAFFR^-/-^ mice are required for the protective immunity induced by WNV E-αCD180 vaccination.

To test whether Abs generated in BAFFR^-/-^ mice are sufficient to confer protection, we passively transferred sera from WNV E-αCD180 vaccinated and infected BAFFR^-/-^ mice into μMT mice. In contrast to sera from naïve BAFFR^-/-^ mice, sera from vaccinated and WNV infected BAFFR^-/-^ or WT mice protected μMT mice from WNV-induced death (Fig 6F). Finally, since we observed that BAFFR^-/-^ mice developed humoral immunity after passive transfer of immune sera and virus challenge, we tested whether our immunization strategy induced long-lived PCs in BAFFR^-/-^ mice. Indeed, we detected WNV E-specific ASCs in the BM 6 months after infection of WNV E-αCD180 vaccinated BAFFR^-/-^ mice (Fig 6G, S4C Fig)., and vaccinated mice survived for over 15 months after WNV challenge. Thus, this novel αCD180-based vaccination method induces long-lasting Ab responses and protective immunity in mature-B cell deficient BAFFR^-/-^ mice by activating Ag-specific humoral immunity in immature B cells.

### αCD180-based immunization induces Ag-specific B cell responses by targeting splenic BAFFR^-/-^ immature B cells

**Fig 7:**
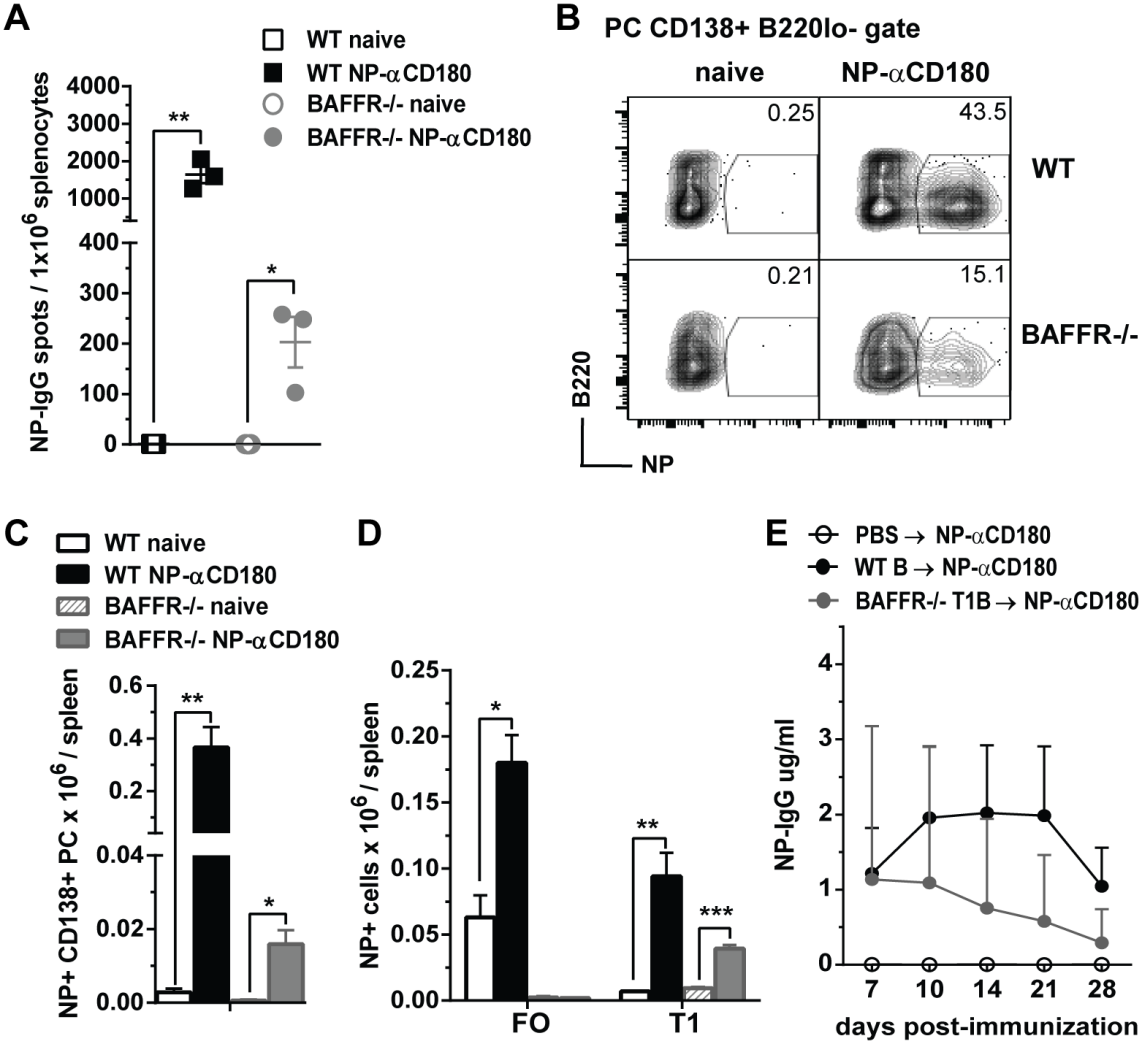
Antigen-specific IgG production after adoptive transfer of splenic WT B cells or BAFFR^-/-^ T1 B cells into μMT mice and immunization with NP-αCD180. *A*-*D*, WT and BAFFR^-/-^ mice were immunized i.v. with 50 ug/mouse of NP-αCD180; 7 days after immunization spleens were harvested from naïve and immunized mice, NP-IgG ASCs were measured by ELISPOT (*A*), and NP-specific cell populations were determined by flow cytometry (*B-D*). In *B* and C, PCs were gated as CD138^+^ B220^lo-^ cells and NP-specific PCs are shown as frequencies in representative dot plots (*B*) and as absolute cell numbers (*C*). In *D*, absolute cell numbers of NP-specific FO and T1 B cells are shown (the gating strategy is shown in S1 Fig). In *A*-*D*, data are from one representative of two independent experiments using three mice/group. In *A*, *C* and *D*, data shown are means ± SEM, statistics were determined by two-tailed Student’s *t* test and significant *p* values are indicated as follows: * *p*<0.05, ** *p*<0.01, *** *p*<0.001. *E*, 20 x 10^6^ purified (see Methods section) total B cells from WT mice and T1 B cells from BAFFR^-/-^ mice or PBS as vehicle control were transferred i.v. into μMT recipients 18 h before i.v. immunization with 100ug/ mouse of NP-αCD180. NP-specific IgG levels were measured by ELISA at the indicated time points post-immunization. Data are from one representative of two independent experiments with 4 recipient mice per group. In *E*, data are shown as means ± SEM and statistics were determined by two-tailed Mann-Whitney *t* test and at the indicated points, a significant *p* value of * *p*<0.05 was found for μMT mice adoptively transferred with WT B cells or BAFFR^-/-^ T1 B cells compared to μMT mice inoculated with PBS.

To examine if αCD180-based immunization could target and activate splenic immature B cells in BAFFR^-/-^ mice, we immunized WT and BAFFR^-/-^ mice with a conjugate comprised of anti-CD180 and the hapten 4-hydroxy-3-nitro-phenacetyl (NP) (NP-αCD180). We used this model Ag because of available reagents including a chromophore conjugated with NP that enabled us to detect Ag-specific ASCs and splenic Ag-specific B cell subsets. NP-αCD180 induced a significant number of splenic NP-IgG ASCs in BAFFR^-/-^ mice 7 days after immunization, although, as expected, at a level lower than in WT mice (Fig 7A). Splenic NP-specific PCs also were induced in BAFFR^-/-^ mice, and again to a lesser extent than in WT mice (Fig 7B and 7C). Neither NP-specific MZ B cells nor T2 B cells were induced in WT or BAFFR^-/-^ mice after NP-αCD180 immunization. In WT mice both splenic NP-specific FO and T1 B cells expanded after immunization with NP-αCD180, whereas in BAFFR^-/-^ mice only NP-specific T1 B cells increased significantly (Fig 7D). These data demonstrate that Ag-αCD180 immunization targets and increases Ag-specific immature B cells in BAFFR^-/-^ mice.

To test whether immature T1 B cells were capable of producing Ag-specific IgGs, we adoptively transferred T1 B cells purified from BAFFR^-/-^ mice into μMT mice, immunized the recipients with NP-αCD180 and measured NP-specific IgG responses (Fig 7E). Notably, NP-αCD180 immunization of μMT mice that received donor BAFFR^-/-^ T1 B cells induced NP-specific IgG albeit at levels that were slightly less than μMT mice that received donor splenic B cells from WT mice (Fig 7E). In conclusion, splenic T1 B cells are capable of responding to Ag after immunization with NP-αCD180 and producing Ag-specific IgG Abs.

## Discussion

Although BAFFR^-/-^ mice deficient in mature B cells succumb to WNV infection, they can be protected against death by either passive or active immunity. Their course of infection was similar to that seen in fully B cell-deficient μMT mice, except that μMT mice had elevated viral loads in sera and the CNS earlier post infection [22]. However, as with μMT mice [22,39], and unlike WT mice, BAFFR^-/-^ mice did not show clearance of WNV from the sera, spleen or brain by day 7–8 post infection. Thus, the WNV-specific humoral immune responses in naïve BAFFR^-/-^ mice were not sufficient to control infection. Although mature B cells are not essential for innate immune responses and viral control at earlier stages of WNV infection, they are required for adaptive immune responses that prevent an uncontrolled infection in the CNS and fatal encephalitis.

Both anti-WNV IgG Abs and nAbs developed in WNV-infected BAFFR^-/-^ mice, but were reduced and delayed compared to Ab levels in WT mice. Indeed, the levels of nAbs at day 8 post-infection in BAFFR^-/-^ mice were not different from levels in WT mice at day 5 post-infection. Unlike μMT mice, after passive administration of immune sera, BAFFR^-/-^ mice survived and went on to develop long-term protective immunity. Consistent with earlier studies [39], μMT mice were protected by immune Abs only in the short term and eventually succumbed to infection. Protected BAFFR^-/-^ mice developed anti-WNV IgG Abs at later time points and at higher levels than that achieved by passive transfer. For WNV, as well as for other encephalitic viruses, the persistence of B cells and virus-specific Ab secreting cells in the CNS are critical to control clinical signs of disease, limit viral persistence, and allow long-term survival, even in the absence of CTL responses [40,41,42]. There were no significant differences in splenic T cells and myeloid cell populations in BAFFR^-/-^ mice vs. μMT mice after WNV infection. Therefore, the ability of BAFFR^-/-^ mice after immune sera transfer to develop WNV-specific immune responses, which sustained a WNV re-challenge, is likely due to the presence of immature B cells and their ability to produce WNV-specific IgG Abs. In support of this idea, Abs induced by vaccination and infection of BAFFR^-/-^ mice when transferred into B-cell deficient μMT mice were sufficient to protect against lethal infection.

While the absence of mature B cells in BAFFR^-/-^ mice most likely is responsible for their susceptibility to WNV infection, a T cell defect could contribute to the reduced immunity. B cells play a major role in splenic T cell zone development and are required for normal numbers of splenic T cells [43]. BAFFR^-/-^ mice are reported to have either normal [9,10] or decreased [44] splenic T cells numbers. We found lower numbers of CD4^+^ and CD8^+^ T cells in BAFFR^-/-^ mice compared to WT mice, prior to and after WNV infection. BAFFR expression on activated/memory T cell subsets can mediate BAFF-dependent co-stimulatory responses [11]. Notwithstanding this observation, BAFFR^-/-^ T cells responded to WNV peptides in vitro and matured into Tef cells in vivo normally.

The newly formed T1 B cells present in the spleen of BAFFR^-/-^ mice appear to contribute to the humoral immune responses to WNV infection. In particular, WNV infection of BAFFR^-/-^ mice did not trigger B cell differentiation into T2, FO or MZ B cells that might be a source of anti-WNV Ab responses. Moreover, WNV challenge did not induce any expansion in splenic B1 B cells or B1 B cells, mature B cells and PCs from the peritoneal cavity of BAFFR^-/-^ mice. After WNV infection, splenic B cells in BAFFR^-/-^ mice still were mostly T1 B cells that retained an immature phenotype, and had expanded in numbers. Only a small number of FO-like B cells accumulated upon infection of BAFFR^-/-^ mice, and these FO-like B cells, unlike in WT mice, retained features of newly formed B cells. Consistent with a key role for T1 B cells during WNV infection, one week after WNV infection of BAFFR^-/-^ mice, when both IgG Abs and nAbs had developed, GC B cells were not detectable. These results agree with the described requirement for BAFFR for B cell transition from T1 cells to a later developmental stage [9,10,45].

A CD180-based immunization strategy that targets Ags directly to B cells can induce strong and persistent humoral immune responses even in immunocompromised mice lacking mature B cells and protect them from lethal WNV infection. Immunization of BAFFR^-/-^ mice with WNV E-αCD180 induced protective immunity and a substantial increase in WNV-specific IgG and nAbs. Previously, we showed that inoculation of NP-αCD180 into BAFFR^-/-^ mice produced as much NP-specific IgG as in WT mice [27]. Thus, Ag targeting to CD180, while requiring B cells, does not appear to require mature B cells. Indeed, we found that NP-αCD180 immunization of BAFFR^-/-^ mice not only induced increases in splenic NP-specific IgG ASCs and PCs, but also induced the expansion of NP-specific T1 B cells. In contrast, in WT mice, both NP-specific FO and T1 B cell numbers were increased. It is known that a second signal can rescue T1 B cells from cell death induced by BCR stimulation alone [46]. Thus, the combination of signaling by WNV E-αCD180 through both the BCR and CD180 on BAFFR^-/-^ T1 B cells induced class switching into WNV E-specific IgG, nAbs, and protection from lethal WNV infection. Indeed, immunization with WNV E alone or anti-CD180 alone did not protect BAFFR^-/-^ mice. The partial rescue we observed when BAFFR^-/-^ were immunized with the control WNV E-IgG2a may be due to the targeting of WNV E protein to other APCs (e.g., Mϕ and DCs) through the Fc domain in the WNV E-iso conjugate, alone or with the help of complement components [24,47]. However, the potency of the control rat IgG2a vaccine was limited by the inability to trigger strong anti-WNVE IgG and nAb responses. The inability of WNV E-αCD180 to rescue μMT mice from lethal WNV infection, confirmed a requirement of B cells for mediating the protective activity of the anti-CD180 based vaccine.

Our data show that immature T1 B cells can contribute to protective immunity against WNV. These results are consistent with studies showing that newly-formed B cells residing in the BM and splenic red pulp (RP) can class-switch and become IgG producing cells [48,49]. Indeed, we and others have found that T1 B cells constitutively express higher levels of activation-induced deaminase (AID) than FO B cells, and can undergo somatic hypermutation and produce IgG in response to a combination of BCR and TLR stimuli [50,51,52,53,54]. In transgenic mice overexpressing TLR7, splenic immature T1 B cells selectively expand, have increased expression of AID, and production of IgG Abs in the RP [55]. This TLR-driven dysregulation of T1 cells can result in production of IgG autoantibodies. Jacobs et al. [56] reported that T1 B cells express TACI, a second receptor for BAFF. TACI^+^ T1 B cells obtained from transgenic mice overexpressing BAFF expressed AID and T-bet and were enriched for class-switched B cells with autoreactivity. Thus, after WNV infection of BAFFR^-/-^ mice, transitional B cells expressing TACI may respond to viral RNA and to BAFF and expand to promote protective immunity. In support of this idea, BAFFR^-/-^ mice, in contrast to BAFF^-/-^ mice, can develop Ab responses mediated through TACI [10,57]. The splenic RP where T1 B cells reside functions as a front line defense against blood-borne viral infections [58]. Furthermore, after immunization, Ag-specific B cells are found in the RP [59]. Early after WNV infection, type I IFN is produced and virus spreads systemically [22,60]. Since type I IFN can upregulate the expression of TLR7 in B cells including immature B cells [33,34], a plausible model is that after WNV infection, type I IFN augments TLR7 expression in T1 B cells, which enables them to be more responsive to RNA-containing WNV entering the RP. The innate RP B cells could respond rapidly to virus and develop into extrafollicular Ab producing cells or Ag presenting cells. The fact that upon WNV infection T1 B cells from BAFFR^-/-^ and WT mice upregulate TLR7 supports this possibility.

When we adoptively transferred BAFFR^-/-^ T1 B cells into μMT mice and immunized them with NP-αCD180, we detected a significant induction of NP-specific IgG in the sera of recipient μMT mice, which lack all other B cells. These data demonstrate that peripheral non-splenic B cells are not necessary for IgG Abs production after CD180-based immunization. It remains possible that a small number of FO-like splenic BAFFR^-/-^ B cells might contribute to this IgG response; however, no NP-specific FO B cells were detected after immunization of BAFFR^-/-^ mice. Furthermore, the levels of WNV-specific IgG and nAbs in immunized and infected BAFFR^-/-^ mice are several orders of magnitude higher than the small increase in FO-like B cells after infection. We conclude that in BAFFR^-/-^ mice immature B cells are the most likely source of the Ag-specific IgG induced after Ag-αCD180 immunization.

Although more study is needed to determine the contribution of humoral responses from immature B cells, our study highlights the possible relevance of an overlooked role of newly-formed B cells in immunocompromised settings. The elderly and immunocompromised individuals are at greater risk for severe viral infections, but also are the most unresponsive populations to vaccination [17,18,61]. Previous studies have shown live-attenuated or inactivated WNV vaccines are effective in stimulating protective immunity in old mice [62,63]. These vaccine formulations often require iterative boosting. Targeting Ags directly to B cells through the CD180 receptor induced protective immunity even in immunocompromised BAFFR^-/-^ mice, and a single immunization of WNV E-αCD180 was sufficient to confer protection. Another aspect of this immunization strategy is the ability to induce durable humoral immunity in immunocompromised BAFFR^-/-^ mice. This finding is consistent with our previous observation that Ag-αCD180 induces immunological memory in WT mice [27].

A recent study on HIV vaccines reported that human naïve B cells can be induced to undergo affinity maturation and produce broadly neutralizing Abs (bnAbs) via a germline targeting strategy [64]. CD180-based immunizations might have the potential to activate immature B cells, which have not yet gone through a selection check point, to produce bnAbs. If so, CD180-based immunization could become a tool to enhance the efficacy of vaccines against viruses like HIV, and to induce protective immunity even in immunocompromised individuals. More studies including the analysis of the variable IgG gene repertoire of nAbs produced by WNV E-αCD180 vaccine in BAFFR^-/-^ mice by are needed to further assess this possibility.

## Materials and methods

### Mice

C57BL/6J mice were purchased from The Jackson Laboratory (Bar Harbor, ME, USA). C57BL/6J BAFFR^-/-^ mice were obtained as a generous gift from Dr. Klaus Rajewsky (Harvard Medical School, Boston, MA). B cell-deficient μMT mice (B6.129S2-*Ighmtm1Cgn*/J) were kindly provided by Dr. David Rawlings (Seattle Children’s Research Institute, Seattle, WA). All mice were housed and maintained in a specific pathogen free facility at the University of Washington.

### Ethics statement

This study was carried out in strict accordance with the recommendations in the Guide for the Care and Use of Laboratory Animals of the National Institutes of Health. All procedures were approved and conducted according to regulations of the Institutional Animal Care and Use Committee of the University of Washington, Seattle, WA (IACUC, Protocol #2242–08). Dissections and footpad injections were performed under anesthesia that was induced and maintained with ketamine hydrochloride and xylazine, and all efforts were made to minimize suffering.

### WNV and infections

All virus stocks were maintained and characterized in within the University of Washington’s NIH-funded Center for Immune Mechanisms of Flavivirus Control. A viral molecular clone generated from plaque-purified lineage 1 WNV-TX02 was used at 10^2^ PFU/dose, as determined by plaque assay on BHK21 cells [28,65].

Female and male 8- to 12-week-old, age- and sex-matched mice were inoculated by s.c. route under anesthesia in the left rear footpad with 10^2^ PFU of WNV-TX in a 20 μl inoculum diluted in a vehicle of HBSS containing 1% fetal bovine serum (FBS). Mice were monitored daily for morbidity and mortality. Clinical scores used were as follows: 1, ruffled fur, lethargy, hunched posture, no paresis; 2, mild paresis involving at least one hind limb; 3, frank paresis involving at least one hind limb and/or conjunctivitis or mild paresis in two hind limbs; 4, severe paresis while still retaining feeling; 5, true paralysis; and 6, moribund. Mice were bled at day 5 and day 8 post-infection and in some experiments at later time points. In some experiments mice were sacrificed at day 7 post-infection and spleens were harvested for flow cytometric analysis (see below).

### Virus burden quantification

Infected mice were euthanized, bled and perfused with 30 ml of phosphate buffered saline (PBS). Whole brain, kidney and spleen were harvested into centrifuge tubes containing 1.4-mm PreCellys ceramic beads (Cayman Chemical, Ann Arbor, MI) and 500 μl of PBS. All tissues were weighed and homogenized in a PreCellys-24 homogenizer (Bertin Technologies, Paris, France) at 845 x g for 20 s. Samples were then centrifuged at 5,000 x g for 10 min, and supernatants were analyzed by a standard plaque assay using BHK cells. Viral titers in sera were determined by real-time qPCR as previously described [29]. Briefly, peripheral blood samples were taken from infected mice at various time points, and viral RNA was extracted using a QiaAMP viral RNA extraction kit (Qiagen, Valencia, CA). Sequences for primers and TaqMan probes used, were as previously published [66].

### WNV-specific antibody analyses

WNV-specific IgM and total IgG levels were determined by an ELISA using a purified recombinant WNV E protein as described [20]. In some experiments the quantities of serum WNV E-specific IgM and IgG between WT mice and BAFFR^-/-^ mice were determined using the endpoint titer determination method [67]. In other experiments, to determine the specific amount of WNV E-specific IgM and IgG an adaptation of a previously described ELISA was used [27]. Briefly, ELISA polystyrene plates were coated with either 2 μg/ml anti–mouse IgG (H+L; Jackson ImmunoResearch) for total Ig, or 5 μg/ml WNV E protein, for WNV E-specific Abs. Serial dilutions of recombinant IgM and IgG (Southern Biotech, Birmingham, AL), were used for the standard curve. Serum samples from WNV infected mice were exposed to UV light for 30 min and incubated at 56°C for 30 min to inactivate virus. Recombinant IgM or IgG and serum samples were diluted in 4% milk casein in PBS containing 0.05% Tween-20, and then incubated on the plate for at least 2 h. Goat anti-mouse IgM and total IgG antibodies conjugated to horseradish peroxidase (Southern Biotech, Birmingham, AL) were added according to the manufacturer’s protocol. Absorbance values were read at 450-nm and 570-nm wavelengths to correct for machine background on a Bio-Rad Micromanager plate reader (version 1.2). WNV E-specific IgM and IgG concentrations were calculated comparing with known dilutions of IgM or IgG.

Serum nAb titers were determined by using a PRNT [22]. Briefly, serum samples from mock or WNV-TX infected mice were diluted in DMEM followed by incubation at 56°C for 30 min to inactivate virus and complement factors. Sera were further diluted in two-fold increments and incubated with 10^2^ PFU of WNV-TX at 37°C for 1 h. Standard plaque assays were performed on BHK21 cells and the dilution at which 50% of plaques were neutralized was determined by comparing the number of plaques formed from WNV-infected sera samples to mock infected sera samples.

ELISPOT assays were performed as previously described [27]. Spots numbers were quantified using a CTL-ImmunoSpot S5 Core Analyzer ELISPOT reader with ImmunoSpot Academic version 5.0 software (Cellular Technology Ltd.).

### Passive antibody transfer experiments

Serum samples were isolated from naive or infected/immune WT mice (sera from day 5, 8, 14, 21 and 28 after infection were pooled), heat-inactivated for 30 min at 56°C, and stored at -80°C. Before pooling the sera for passive transfer experiments, an aliquot was reserved for confirming levels of WNV-specific IgM and IgG and neutralizing Ab titers. For passive transfer experiments, mice were administered 0.5 ml of sera i.p. 1 day prior to and 1 day after inoculation with 10^2^ PFU of WNV-TX. Mice then were monitored for morbidity and mortality for up to 4 months. Sera were harvested at the indicated time points to monitor levels of WNV-specific IgM and IgG. In some cases, surviving mice were re-challenged with WNV-TX 10^2^ PFU at day 65 post-infection, and mice were monitored for morbidity and mortality for up to 12 additional months.

### Immunizations with WNV E—anti-CD180 (WNV E-αCD180) conjugates

For immunization experiments, the anti-CD180 (RP/14) hybridoma was kindly provided by K. Miyake (University of Tokyo, Tokyo, Japan), and the rat IgG2a isotype control (9D6) hybridoma was a gift from R. Mittler (Emory University, Atlanta, GA). To ensure equivalence, these mAbs were sequentially purified on the same protein G column and tested for endotoxin by LAL gel-clot assays in GlucaShield buffer (Associates of Cape Cod). Samples were rejected if endotoxin levels were >0.025 EU/mg protein. The RP/14 anti-CD180 mAb (αCD180) or 9D6 rat IgG2a (iso) were conjugated with purified WNV E protein [68] as previously described for conjugation of mAbs with Ags [27,69]. Final WNV E-mAb conjugation ratios were determined by electrophoresis and ELISA as previously described [27]. 20 μg WNV E-αCD180, the amount of conjugate administered to mice in immunization experiments, contains 10 μg WNV E and 10 μg αCD180. For immunizations, mice were inoculated i.v. or i.p. with 200 μl of PBS containing 20 μg/mouse of WNV E-αCD180- or rat -WNVE-IgG2a (WNVE-iso) conjugate. We found no significant difference in the Ab responses when conjugates were administered i.v. or i.p. Mice were bled at day 7, 14, 21 and 28 after immunization. 30 days after immunization mice were infected with 10^2^ PFU of WNV-TX. Mice were bled at day 5 and day 8 post-infection and, in some experiments, at later time points.

### WNV epitope-specific peptides stimulation *in vitro* and major histocompatibility complex (MHC) class I and MHC class II tetramers

For *in vitro* restimulation, 1 μM of the CD8^+^ T cell-specific NS4B 9-mer SSVWNATTA [70] peptide or 1 μM of the CD4^+^ T cell-specific WNV E_641-655_ 15-mer PVGRLVTVNPFVSVA peptide [71] (Genemed Synthesis Inc., San Antonio, TX) was added to 3–15 x 10^6^ splenocytes cultured in RPMI 1640 supplemented with 10% FBS containing 3μg/ml Brefeldin A (BD Biosciences, San Diego, CA) at 37°C for 6 h. Cells were centrifuged and used for intracellular cytokine staining as described below. The D^b^ NS4B MHC class I tetramer was generated in our laboratory as previously described [67]. The I-A^b^ E MHC class II tetramer and CLIP negative control tetramer were generated at the NIH Tetramer Core Facility (Emory University Vaccine Center, Atlanta, GA). Splenocytes harvested from WT and BAFFR^-/-^ mice prior to or 7 days after WNV infection were incubated with the MHC class I tetramer, the MHC class II tetramer or with the CLIP negative control tetramer for 45 min at room temperature (RT) followed by staining for T cell surface markers as described below.

### B cell adoptive transfer and immunizations with NP-αCD180 conjugates

For the adoptive transfer of B cells into μMT mice, splenic B cells from naïve WT or BAFFR^-/-^ were isolated by negative selection enrichment (STEMCELL Technologies, Vancouver, BC, Canada). Highly purified B cells were obtained by both WT and BAFFR^-/-^ mice, as 98% of B cells purified from naïve BAFFR^-/-^ mice were T1 B cells. 2 × 10^7^ purified total B cells from WT mice and T1 B cells from BAFFR^-/-^ mice or PBS as vehicle control were transferred i.v. into μMT recipients 18 h before immunization. The hapten 4-hydroxy-3-nitro-phenacetyl (NP) was conjugated to anti-CD180 (NP-αCD180) as previously reported [27]. The adoptively transferred mice were inoculated i.v. with 200 μl of PBS containing 100 μg/mouse of NP-αCD180. For direct immunizations of WT and BAFFR^-/-^ mice, mice were inoculated i.v. with 50 μg/mouse of NP-αCD180. ELISA and ELISPOT assays to detect NP-IgG in the serum and NP-IgG ASCs in the spleens were performed as previously described [27]. To detect NP-specific cell subsets by flow cytometry NP-PE was prepared by conjugation of NPOsu (Biosearch Technologies) to phycoerythrin (Sigma-Aldrich, St. Louis, MO) as described [27].

### Cell isolation and flow cytometric analysis

Spleens and peritoneal exudate cells (PECs) from naïve or WNV-infected mice at the indicated time points were harvested and dissociated into single cell suspensions as previously described [72]. After erythrocytes lysis, splenocytes and PECs from peritoneal lavage were filtered and processed for staining for flow cytometry. Cells were incubated with Aqua Live-Dead Fixable Dead Cells staining kit (Molecular Probes, Life Technologies, Waltham, MA) protected from light for 20 min at 4°C. Subsequently, cells were incubated with anti-CD16/CD32 blocking Ab (2.4G2) for 10 min at RT and then stained with various Ab mixtures at 4°C. Cells were stained with mAbs conjugated to FITC, PE, allophycocyanin, eFluor450, allophycocyanin-eFluor780, PerCPCy5.5, PE-Cy7, AlexaFluor647, BUV395, BV605, BV421, BV711 and BV650. For analysis of splenic B cell subsets (gating strategy in S1A Fig) according to Gilitay et al. [55], and PECs B cells according to Giordano et al. [72,73], seven- or eight-colors flow cytometry was performed using combinations of mAbs against B220 (RA3-6B2), CD38 (90), GL7 (GL7), CD24 (M1/69) and CD138 (281–2) from BD Horizon/Biosciences (San Jose, CA, USA); IgM (II/41), CD69 (H1.2F3) and CD5 (53–7.3) from eBioscience, (San Diego, CA, USA); CD21/CD35 (7E9), IgD (11-26c.2a), CD93 (AA4.1) and CD23 (B3B4) from BioLegend (San Diego, CA, USA). For analysis of other myeloid cell subsets (gating strategy in S2 Fig), nine- to eleven-colors flow cytometry was performed using combinations of mAbs against: CD19 (1D3), CD11b (M1/70), CD11c (N418) from eBioscience (San Diego, CA, USA); B220 (RA3-6B2) and Ly6C (AL-21) from BD Horizon/Biosciences (San Jose, CA, USA); CD3 (17A2), NK1.1 (PK136), CD8α (53–6.7), Ly6G (1A8), F4/80 (BM8) and CD169 (3D6.112) from BioLegend (San Diego, CA, USA). Splenic myeloid cell subsets were defined as described previously [72] with some modifications (gating strategy in S3 Fig). After gating out B cells and T cells (CD19^-^CD3^-^) cell populations were phenotyped as follows: NK cells, NK1.1^hi^CD11b^int^; neutrophils (Nph), CD11b^hi^Ly6G^hi^Ly6C^int^SSC^int-^NK1.1^-^; eosinophils (Eosph), CD11b^hi^SSC^hi^Ly6G^lo^Ly6C^int^NK1.1^-^; Ly6C^hi^ monocytes (Ly6C^hi^ MO), CD11b^hi^Ly6C^hi^CD11c^-^SSC^-^Ly6G^-^NK1.1^-^; Ly6C^hi^ dendritic cells (Ly6C^hi^ DC, CD11b^hi^Ly6C^hi^CD11c^hi^ SSC^-^Ly6G^-^NK1.1^-^; Ly6C^lo^ MO, CD11b^int^CD11c^-^Ly6C^lo^SSC^-^Ly6G^-^NK1.1^-^; plasmacytoid DCs (pDC), CD11b^-^CD11c^lo^B220^+^Ly6G^-^NK1.1^-^; CD8^+^ cDCs, CD11c^hi^CD8^+^B220^-^Ly6G^-^NK1.1^-^; CD8^-^ cDCs, CD11c^hi^CD8^-^B220^-^Ly6G^-^NK1.1^-^; red pulp macrophages (RPM), F4/80^+^SSC^hi^CD11b^+^CD11c^lo^Ly6G^-^NK1.1^-^; marginal zone macrophages (MZM), CD169^+^F4/80^-^CD11b^+^CD11c^lo^Ly6G^-^NK1.1^-^).

For analysis of T cell populations, seven- to eight-colors flow cytometry was performed using combinations of mAbs against: CD3 (145-2C11), CD4 (RM4-5), CD62L (MEL-14) from eBioscience (San Diego, CA, USA); CD8α (53–6.7), CD44 (IM7), interferon (IFN)γ (XMG1.2) and TNFα (MP6-XT22) from BioLegend (San Diego, CA, USA). T cells subsets were defined as previously described [74]: CD4^+^ T cells, CD3^+^CD4^+^CD8^-^; CD8^+^ T cells, CD3^+^CD4^-^CD8^+^; CD4^+^ T naïve (CD4 Tn), CD3^+^CD4^+^ CD8^-^CD62L^+^CD44^lo-^); CD4^+^ T effector (CD4 Teff), CD3^+^CD4^+^ CD8^-^CD62L^-^CD44^hi^; CD4^+^ T central memory (CD4 Tcm), CD3^+^CD4^+^ CD8^-^CD62L^+^CD44^+^; CD8^+^ T naïve (CD8 Tn), CD3^+^CD4^-^CD8^+^CD62L^+^CD44^lo-^); CD8^+^ T effector (CD8 Tef), CD3^+^CD4^-^CD8^+^CD62L^-^CD44^hi^; CD8^+^ T central memory (CD8 Tcm), CD3^+^CD4^-^CD8^+^CD62L^+^CD44^+^.

For intracellular staining cells were stained with mAbs for surface markers, fixed and permeabilized using 0.1% saponin in staining buffer (2% FBS in PBS) followed by anti-IFNγ or anti-TNFα staining for 20 min at RT. Cells were processed on an LSRII FACScan analyzer (Becton Dickinson, Franklin Lakes, NJ, USA) using FACSDiva software and data analysis was performed with FlowJo software.

### Statistical analysis

All statistical analysis was performed with Prism software (GraphPad Software, Inc.). A *p* value of <0.05 was considered significant. For flow cytometry experiments and ELISPOT assays a two-tailed unpaired t-test was performed for all experiments comparing two groups. For experiments involving detection of serum Ab levels the two-tailed Mann-Whitney nonparametric test was performed for all experiments comparing two groups. Statistical analysis of flow cytometry experiments and ELISPOT assays when comparing more than two groups was performed with One-Way ANOVA corrected for multiple comparisons using the Holm-Sidak method. For experiments involving detection of serum Abs levels and comparing more than two groups, Kruskal-Wallis corrected with the Dunn’s multiple comparisons post-test was used. For survival studies, the log-rank Mantel-Cox test was used. For comparison of time courses of clinical scores or mouse weights, significance was determined with t-tests corrected for multiple comparisons using the Holm-Sidak method.

## Supporting information

S1 Fig Analyses of splenic and peritoneal cavity B cell subsets from WT and BAFFR^-/-^ mice after WNV infection. *A-D*, splenocytes and *E*, peritoneal exudate cells (PECs) from naïve and WNV-infected (day 7) WT and BAFFR^-/-^ mice. *A*-*E*, Debris, doublets and nonviable cells were excluded from total splenocytes and PECs. *A*, Shows the gating strategy to identify mouse splenic B cell subsets, B220^+^ cells (B cells) were subdivided in B cell subsets defined based on their expression of CD21 and CD24 (*upper panel*). FO B cells were defined as B220^+^CD21/35^int^CD24^lo^CD23^+^, and T1 B cells as B220^+^CD21^lo/-^CD24^hi^CD23^-^. CD21^hi^CD24^hi^ cells (MZ-T2) were further characterized based on their expression of CD21 and CD23 (*lower panel*) into MZ B cells as B220^+^CD21^hi^CD24^hi^CD23^-^, and T2 B cells as B220^+^CD21^hi^CD24^hi^CD23^+^. An additional B cell subset was defined as CD21^-^CD24^-^ B cells (*upper panel*). *B*, CD93 (AA4.1) expression on T1 and FO B cells from WT mice (*dark grey*) and BAFFR^-/-^ mice (*light grey*). *C*, Frequencies of B220^lo^CD138^+^ PCs in CD21^-^CD24^-^ B cells in representative dot plots from three independent experiments. *A*-*C*, show representative dot plots from three independent experiments with 3 mice per group. *D*, Splenic B1 B cells were defined as B220^lo-^CD19^hi^CD23^-^IgM^hi^IgD^lo^ cells, and as expected 80–90% of B1 B cells in the spleen were CD5^+^. In *D* the graph shows means ± SEM of absolute numbers and summarizes data from two independent experiments (N = 6–7 mice). *E*, *left panel* PEC B cells were defined as B220^+^CD19^+^ (CD5^-^CD23^+^IgM^lo^IgD^hi^) B cells and B1 B cells B220^lo-^CD19^hi^CD23^-^IgM^hi^IgD^lo^, subsequently subdivided in CD5^+^ B1a B cells and CD5^-^ B1b cells. *E*, *right panel* shows B220^lo^CD138^+^ PCs in peritoneal exudate. *E*, shows means ± SEM of absolute numbers from a representative of two independent experiments, each performed with 3–4 mice per group. In *D* and *E* statistics were determined by one way-ANOVA corrected with Holm-Sidak for multiple comparisons post-test; ** *p*<0.01, *** *p*<0.001, **** *p*<0.0001.
(PDF)

S2 Fig Gating strategy to identify mouse splenic myeloid cell populations. Splenocytes from WT mice 7 days after WNV infection are shown. Shown are single, live cells gated on CD19^-^CD3^-^NK1.1^-^ cells (non-B, T, NK cells) and myeloid populations were defined based on their relative expression of CD11b vs. CD11c and further subdivided in other populations (see Methods for details). Conventional DCs (cDCs) were gated on CD11c^hi^CD11b^int-^ and subdivided into CD8^+^ cDCs and CD8^-^ cDCs. The CD11b^hi^ gate based on Ly6G expression and SSC levels was subdivided into neutrophils (Nphs) CD11b^hi^SSC^int^Ly6G^hi^ and monocytes (MOs), CD11b^hi^SSC^lo^Ly6G^lo-^. The MO gate was subdivided in Ly6C^lo^ MOs and Ly6C^hi^ monocytes/dendritic cells (Ly6C^hi^ MO/DC). The Ly6C^hi^ MO/DC population was further subdivided into Ly6C^hi^ MOs (CD11c^lo-^) and Ly6C^hi^ DCs (CD11c^+^).
(PDF)

S3 Fig Comparison of T cell and myeloid populations in BAFFR^-/-^ and μMT mice. Spleens from naïve (*A*) or WNV-infected for 7 days (*B*) BAFFR^-/-^ or μMT mice were harvested and cell populations were determined by flow cytometry. *A* and *B*, for definitions of cell populations see Methods and S2 Fig. Graphs show means ± SEM of total cell numbers from one experiment using three mice/group. Statistics were determined by two-tailed Student’s *t* test * *p*<0.05, ** *p*<0.01, *** *p*<0.001.
(PDF)

S4 Fig WNV E-αCD180 immunization enhances WNV E-specific IgG in BAFFR^-/-^ mice, and in contrast to WNV E protein or αCD180 alone, protects BAFFR^-/-^ mice from lethal WNV challenge. *A*, WT and BAFFR^-/-^ mice were inoculated i.v. with 20 μg/mouse WNV E-αCD180. Anti-WNV E IgG levels were measured by ELISA at the indicated time points post-immunization. Data are pooled from two independent experiments using WT (N = 6) and BAFFR^-/-^ mice (N = 12). Statistics were determined by two-tailed Mann-Whitney *t* test, * *p*<0.01. *B*, BAFFR^-/-^ mice were inoculated i.v. with 10 μg/mouse WNV E protein (WNV Ep) only, 10 μg/ml anti-CD180 (αCD180) or 20 μg/mouse WNV E-αCD180 and 30 days prior to s.c. infection with 10^2^ PFU of WNV. Survival data are pooled from two independent experiments (N = 10). Statistics were performed using a log rank test, comparing all groups to BAFFR^-/-^ mice inoculated with WNV E-αCD180, * *p*<0.01. *C* shows examples of images of long-lived ASCs detected by WNV E-IgG ELISPOT assay on BMs of BAFFR^-/-^ mice 6 months after WNV infection of WNV E-αCD180 vaccinated mice (Fig 6G).
(PDF)
