# Supplementary material for: Protection of mice deficient in mature B cells from West Nile virus infection by passive and active immunization
Source: PLoS Pathog. 2017 Nov 27;13(11):e1006743. doi: 10.1371/journal.ppat.1006743 (PMC5720816; doi:10.1371/journal.ppat.1006743)
Supplement: S1 Fig — A-D, splenocytes and E, peritoneal exudate cells (PECs) from naïve and WNV-infected (day 7) WT and BAFFR-/- mice. A-E, Debris, doublets and nonviable cells were excluded from total splenocytes and PECs. A, Shows the gating strategy to identify mouse splenic B cell subsets, B220+ cells (B cells) were subdivided in B cell subsets defined based on their expression of CD21 and CD24 (upper panel). FO B cells were defined as B220+CD21/35intCD24loCD23+, and T1 B cells as B220+CD21lo/-CD24hiCD23-. CD21hiCD24hi cells (MZ-T2) were further characterized based on their expression of CD21 and CD23 (lower panel) into MZ B cells as B220+CD21hiCD24hiCD23-, and T2 B cells as B220+CD21hiCD24hiCD23+. An additional B cell subset was defined as CD21-CD24- B cells (upper panel). B, CD93 (AA4.1) expression on T1 and FO B cells from WT mice (dark grey) and BAFFR-/- mice (light grey). C, Frequencies of B220loCD138+ PCs in CD21-CD24- B cells in representative dot plots from three independent experiments. A-C, show representative dot plots from three independent experiments with 3 mice per group. D, Splenic B1 B cells were defined as B220lo-CD19hiCD23-IgMhiIgDlo cells, and as expected 80–90% of B1 B cells in the spleen were CD5+. In D the graph shows means ± SEM of absolute numbers and summarizes data from two independent experiments (N = 6–7 mice). E, left panel PEC B cells were defined as B220+CD19+ (CD5-CD23+IgMloIgDhi) B cells and B1 B cells B220lo-CD19hiCD23-IgMhiIgDlo, subsequently subdivided in CD5+ B1a B cells and CD5- B1b cells. E, right panel shows B220loCD138+ PCs in peritoneal exudate. E, shows means ± SEM of absolute numbers from a representative of two independent experiments, each performed with 3–4 mice per group. In D and E statistics were determined by one way-ANOVA corrected with Holm-Sidak for multiple comparisons post-test; ** p<0.01, *** p<0.001, **** p<0.0001. (PDF) [file ppat.1006743.s001.pdf]

S1 Fig.

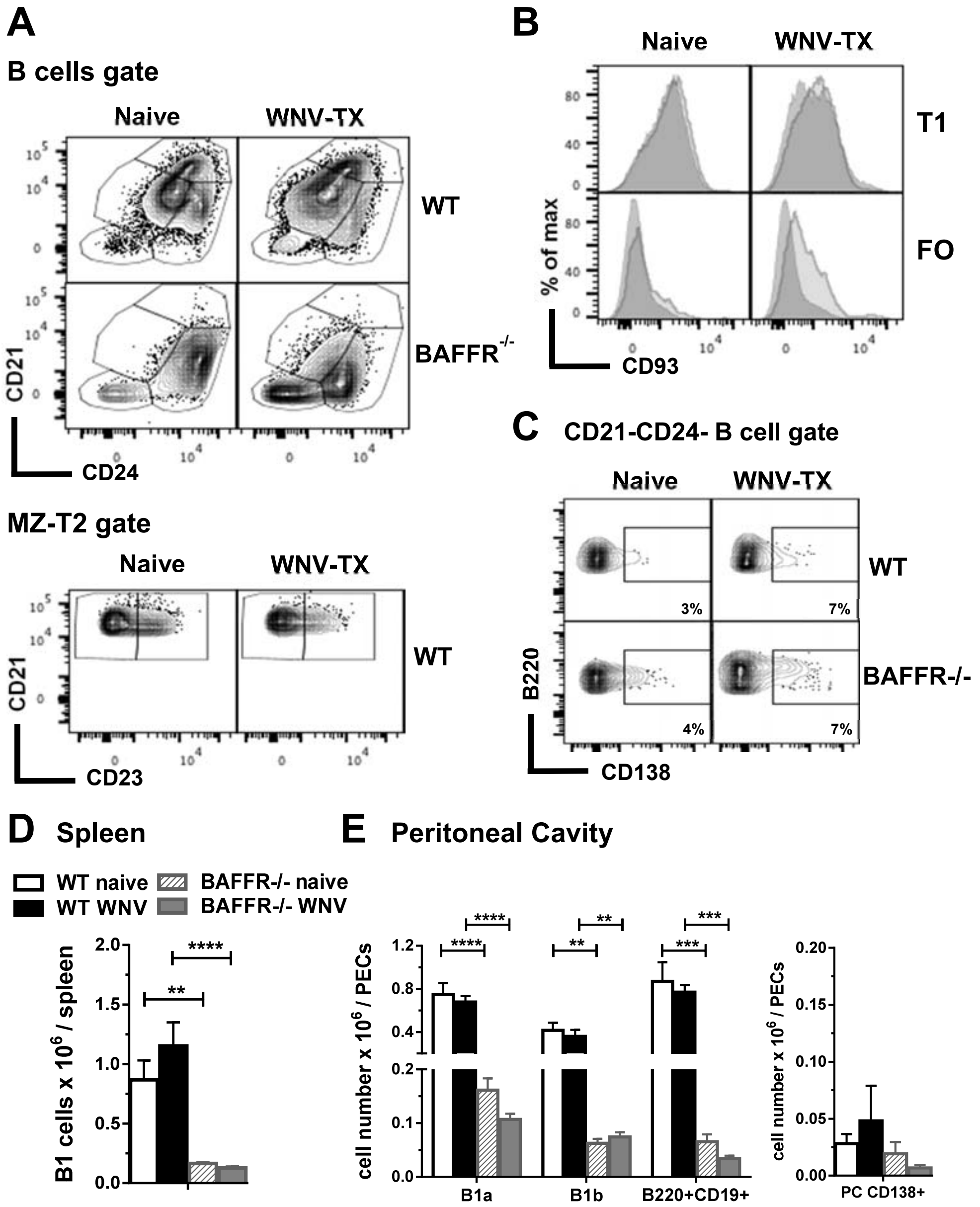

A
B cells gate
Naive
WNV-TX
WT
BAFFR-/-
CD21
CD24
MZ-T2 gate
Naive
WNV-TX
WT
CD21
CD23
B
Naive
WNV-TX
T1
FO
% of max
CD93
C
CD21-CD24- B cell gate
Naive
WNV-TX
WT
BAFFR-/-
3%
7%
4%
7%
B220
CD138
D
Spleen
WT naive
WT WNV
BAFFR-/- naive
BAFFR-/- WNV
B1 cells x 10^6 / spleen
E
Peritoneal Cavity
cell number x 10^6 / PECs
B1a
B1b
B220+CD19+
PC CD138+
