# Supplementary figures and images for: Protection of mice deficient in mature B cells from West Nile virus infection by passive and active immunization
Source: PLoS Pathog. 2017 Nov 27;13(11):e1006743. doi: 10.1371/journal.ppat.1006743 (PMC5720816; doi:10.1371/journal.ppat.1006743)

## S2 Fig.

### Spleen

Debris, doublets, dead cells excluded

CD19-CD3-NK1.1- gate

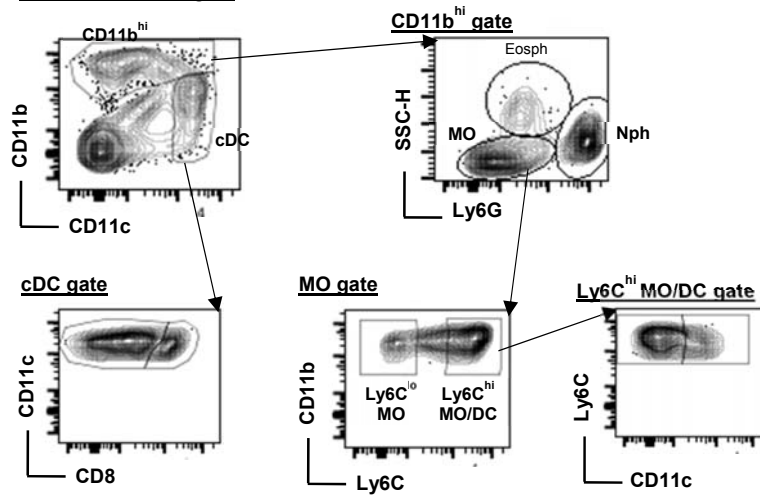

Supplement: S2 Fig — Splenocytes from WT mice 7 days after WNV infection are shown. Shown are single, live cells gated on CD19-CD3-NK1.1- cells (non-B, T, NK cells) and myeloid populations were defined based on their relative expression of CD11b vs. CD11c and further subdivided in other populations (see Methods for details). Conventional DCs (cDCs) were gated on CD11chiCD11bint- and subdivided into CD8+ cDCs and CD8- cDCs. The CD11bhi gate based on Ly6G expression and SSC levels was subdivided into neutrophils (Nphs) CD11bhiSSCintLy6Ghi and monocytes (MOs), CD11bhiSSCloLy6Glo-. The MO gate was subdivided in Ly6Clo MOs and Ly6Chi monocytes/dendritic cells (Ly6Chi MO/DC). The Ly6Chi MO/DC population was further subdivided into Ly6Chi MOs (CD11clo-) and Ly6Chi DCs (CD11c+). (PDF) [file ppat.1006743.s002.pdf]

# S3 Fig.

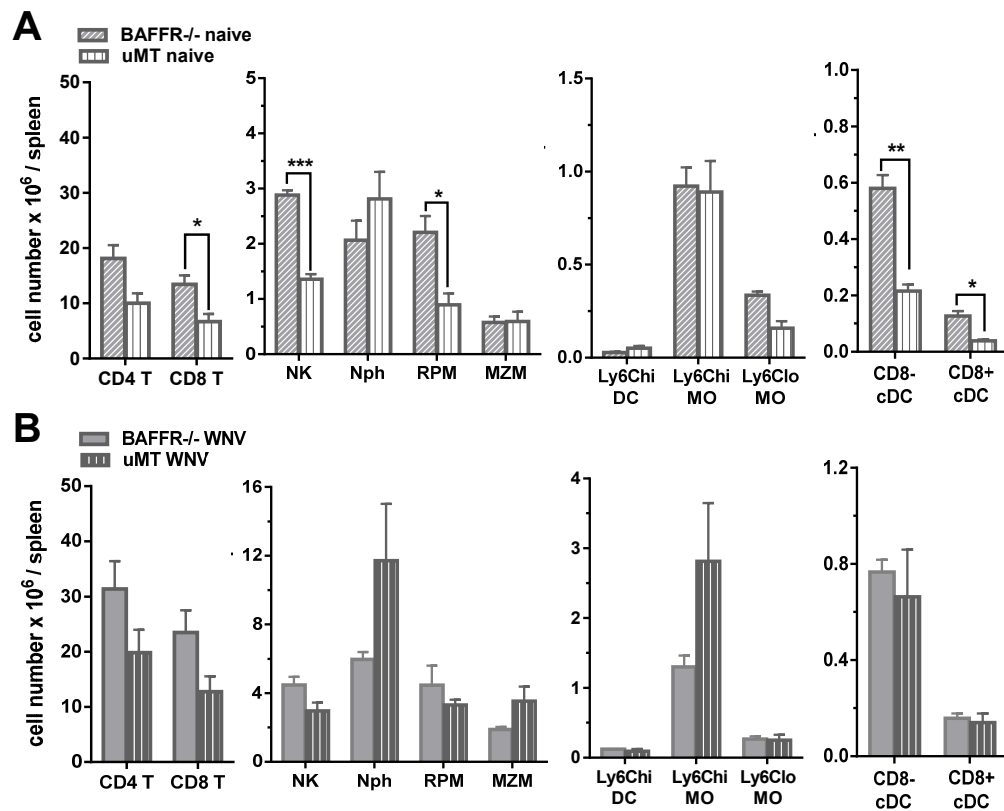

Supplement: S3 Fig — Spleens from naïve (A) or WNV-infected for 7 days (B) BAFFR-/- or μMT mice were harvested and cell populations were determined by flow cytometry. A and B, for definitions of cell populations see Methods and S2 Fig. Graphs show means ± SEM of total cell numbers from one experiment using three mice/group. Statistics were determined by two-tailed Student’s t test * p<0.05, ** p<0.01, *** p<0.001. (PDF) [file ppat.1006743.s003.pdf]

S4 Fig.

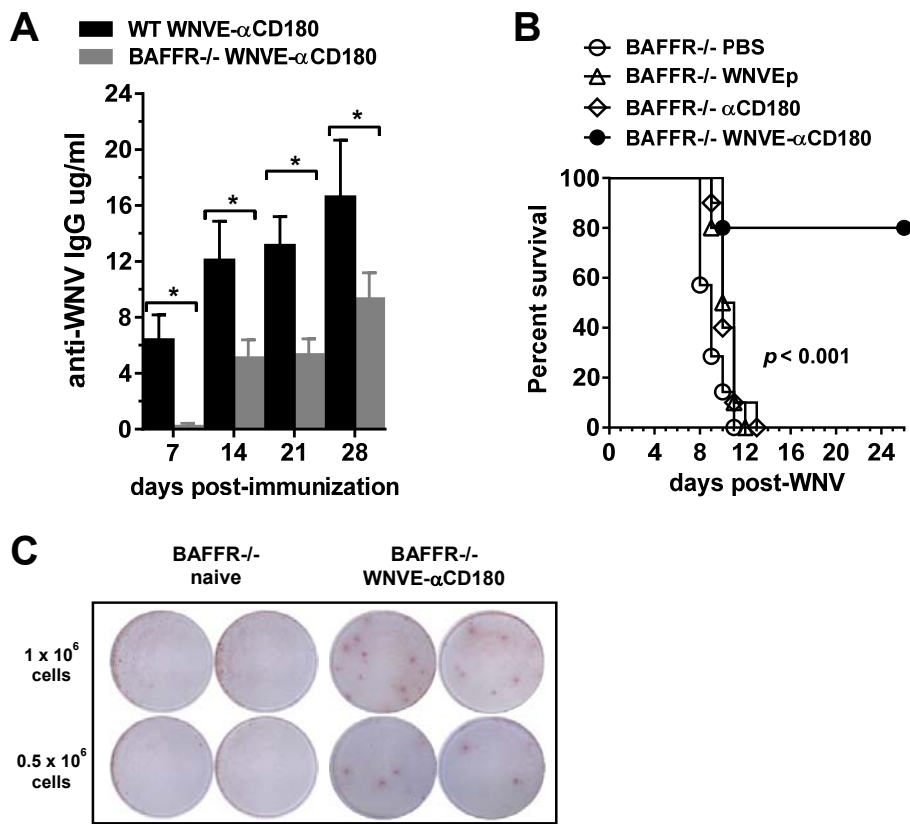

Supplement: S4 Fig — A, WT and BAFFR-/- mice were inoculated i.v. with 20 μg/mouse WNV E-αCD180. Anti-WNV E IgG levels were measured by ELISA at the indicated time points post-immunization. Data are pooled from two independent experiments using WT (N = 6) and BAFFR-/- mice (N = 12). Statistics were determined by two-tailed Mann-Whitney t test, * p<0.01. B, BAFFR-/- mice were inoculated i.v. with 10 μg/mouse WNV E protein (WNV Ep) only, 10 μg/ml anti-CD180 (αCD180) or 20 μg/mouse WNV E-αCD180 and 30 days prior to s.c. infection with 102 PFU of WNV. Survival data are pooled from two independent experiments (N = 10). Statistics were performed using a log rank test, comparing all groups to BAFFR-/- mice inoculated with WNV E-αCD180, * p<0.01. C shows examples of images of long-lived ASCs detected by WNV E-IgG ELISPOT assay on BMs of BAFFR-/- mice 6 months after WNV infection of WNV E-αCD180 vaccinated mice (Fig 6G). (PDF) [file ppat.1006743.s004.pdf]
